# Sucrose-phosphate phosphatase from sugarcane reveals an ancestral tandem duplication

**DOI:** 10.1186/s12870-020-02795-5

**Published:** 2021-01-07

**Authors:** Vania Gabriela Sedano Partida, Henrique Moura Dias, Diana Susana Martinez Corcino, Marie-Anne Van Sluys

**Affiliations:** Departamento de Botânica IB, USP, São Paulo, SP 05508-090 Brazil

**Keywords:** Sucrose metabolism, *Saccharum*, Comparative genomics, Gene expression, Phylogenetic inference, Poaceae

## Abstract

**Background:**

Sugarcane is capable to store large amounts of sucrose in the culm at maturity hence it became a major source of sucrose for the food and the renewable energy industries. Sucrose, the main disaccharide produced by photosynthesis, is mainly stored in the vacuole of the cells of non-photosynthetic tissues. Two pathways are known to release free sucrose in plant cells, one is de novo synthesis dependent on sucrose phosphate synthase (SPS) and sucrose phosphate phosphatase (S6PP) while the other is regulatory and dependent on sucrose synthase (SuSy) activity. The molecular understanding of genes that give rise to the expression of the enzyme sucrose phosphate phosphatase, responsible for the release of sucrose in the last synthetic step lag behind the regulatory *SuSy* gene.

**Results:**

Sugarcane genome sequencing effort disclosed the existence of a tandem duplication and the present work further support that both *S6PP.1* and *S6PP_2D* isoforms are actively transcribed in young sugarcane plants but significantly less at maturity. Two commercial hybrids (SP80–3280 and R570) and both *Saccharum spontaneum* (IN84–58) and *S.officinarum* (BADILLA) exhibit transcriptional activity at three-month-old plants of the tandem *S6PP_2D* in leaves, culm, meristem and root system with a cultivar-specific distribution. Moreover, this tandem duplication is shared with other grasses and is ancestral in the group.

**Conclusion:**

Detection of a new isoform of S6PP resulting from the translation of 14 exon-containing transcript (*S6PP_2D*) will contribute to the knowledge of sucrose metabolism in plants. In addition, expression varies along plant development and between sugarcane cultivars and parental species.

## Background

Sugarcane economic value as a world commodity is due to its high sugar yield, renewable energy and other biomolecules production [1]. The high sugar yield results from its ability to store large amounts of sucrose in the internodes of the culm as part of its developmental program. Sucrose is the most used form of sugar in plants and its partitioning depends on finetuning synthesis, storage and metabolic uses. Sucrose metabolism can be divided in three steps: synthesis, transport and accumulation. Sucrose synthesis occurs in leaf cells as part of photosynthesis, being the main metabolite obtained in this cellular process. Sucrose is synthesized in the cytosol and released by the action of the enzyme Sucrose-phosphate phosphatase (EC 3.1.3.24, PF 08472), also known as sucrose-6 phosphate phosphohydrolase (S6PP). S6PP catalyzes the last reaction step of this metabolic pathway where sucrose-6-phosphate (Suc6P), product of the enzymatic reaction of sucrose phosphate synthase enzyme (EC 2.4.1.14) (SPS), is dephosphorylated and released as sucrose [2, 3].

The release of free sucrose in the cytosol drives its transport to the phloem conducting vessels, being the most common form of carbohydrate translocated from source tissues to sink tissues and organs of the whole plant [4, 5]. Finally, the produced sucrose is stored in the vacuoles of the parenchyma cells of all the non-photosynthetic tissues. At this stage, accumulation, the sucrose enters the cells by either the apoplastic or symplastic route where the enzyme Sucrose synthase (EC 2.4.1.13) (*SuSy*), plays a regulatory role converting it into UDP-glucose according to the needs of the plant physiology through a reversible reaction. Alternatively, sucrose can also be activated by the SPS to form the intermediate Suc6P which becomes dephosphorylated by the action of the enzyme S6PP [6]. Dephosphorylation at sink tissues provides the energy necessary for the entry of sucrose into the vacuole, where it is finally accumulated in the form of glucose and fructose [7]. Different to this energetic function depicted in most land plants, some cyanobacteria synthesize sucrose as an adaptation to osmotic stress [8].

Modern varieties of sugarcane are interspecific hybrids (*Saccharum* spp.) selected after cross-breeding within *Saccharum* species, mainly between *Saccharum officinarum* L. and *S. spontaneum* L. [9]. Examples of these modern cultivars are R570 (bred in the French island of Reunion) and SP80–3280 (bred in São Paulo, Brazil) and both have their genomes partially sequenced [10, 11]. A previous work released a collection of 314 sugarcane *Bacterial Artificial Chromosomes* (BACs) from R570 cultivar in which a tandem duplication of the gene *S6PP* was described [12]. This tandem duplication was further validated in the recent releases of genomes sequences [10, 13]. The two S6PP genes have a similar gene structure with eight exons, five of which are conserved in size and contain the catalytic site of the protein. Being separated by an intergenic region of 630 bases between the STOP codon of *S6PP.1* and the ATG codon from *S6PP.2* (Figure S1).

The S6PP enzyme is found in vascular (liver plants, gymnosperms and angiosperms) and non-vascular plants (green algae, mosses). In some vascular plants, the S6PP enzyme has been described as a homodimer of about 120 KDa. As examples: Rice S6PP has been described as dimeric of 100–120 KDa, with 50 KDa monomer [14, 15]; the pea S6PP forms also a dimer of 120 KDa, with 55 KDa monomers [16]; whereas *Arabidopsis* S6PP dimer is of 90 KDa, with 52 KDa monomers [17]. Maize S6PP information is only available from denaturing gel and presents molecular mass of 47.2 KDa. *Synechocystis* and *Anabaena*, both cyanobacteria, present a smaller (27–28 KDa) and monomeric S6PP enzyme [8, 18–20]. Despite this monomeric characterization in bacteria, especially belonging to the cyanobacteria clade, a two-domain containing protein SPS and S6PP has been identified in the proteobacteria *Methylobacillus flagellatus*, characterized to be a tetramer of 336 KDa with 84KDa monomers [21]. Regardless of these different S6PP arrangements, the basic unit (monomer) in all species share significant similarity at the amino acid sequence mostly in the catalytic domain [8, 15].

The main aim of the present work is to characterize at molecular level the sugarcane S6PP to contribute to the sucrose metabolism knowledge in plants. We explored the existence of a new isoform, derived from the alternative splicing of the tandem duplication as a monomer with two catalytic domains. In addition to *S6PP.1* and/or *S6PP.2* genes transcripts, we detected a new isoform resulting from the translation of 14 exon-containing transcript (*S6PP_2D*). We demonstrate that both are actively transcribed in different sugarcane tissues, with differential expression along plant development stage and between sugarcane cultivars or parental species.

## Results

### S6PP genomic organization of two commercial sugarcane cultivars

**Fig. 1:**
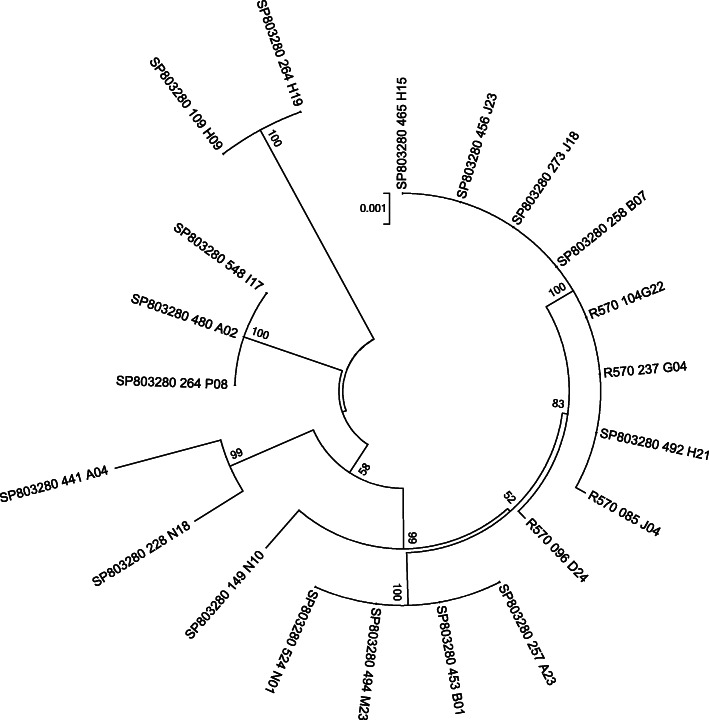
Phylogeny of *S6PP* tandem region in BACs of SP80–3280 and R570 sugarcane hybrids. Maximum Likelihood phylogenetic bootstrap consensus tree inferred bye MEGA7 using the *S6PP* tandem region alignment. Built by substitution *T92 + G* as the best model highest ranked and 1000 bootstraps pseudo-replicates was established for this analysis

A comparative genomic approach addressed the presence of the previously described tandem duplication in two BAC in a collection of 21 sequenced genomic fragments from two sugarcane cultivars, SP80–3280 and R570 [11, 12]. According to Table 1, the tandem duplication is present in all sequenced fragments irrespective of the cultivar. In addition, the genomic environment support that these genes are located at a single locus with structural genomic variants shared between the two cultivars as denoted by their neighboring genes and transposable elements insertions as shown in supplementary Figure S2. To improve our knowledge on the evolutionary history of these genomic fragments, a phylogenetic tree using at least 5000 bases per BAC was built from the alignment of these two tandem genes starting at the ATG from *S6PP.1* up to the stop codon from *S6PP.2* gene. Figure 1 depicts that differences between the genomic regions of these two hybrid cultivars do not group them distinctively. Ten of the SP80–3280 BACs genomic regions branch in one consistent sequence clade with the four R570 genomic regions, while the other seven genomic regions group separately. Grouping of both sugarcane cultivars in one clade is supported with high bootstrap which is indicative that these genomic regions are very similar with a few INDELS and SNPs (data not shown).

**Table 1:** List of genomic fragments identified in 21 BACs containing *S6PP* genes

Sugar cane variety	BAC name	BAC size (bp)	Genomic tandem region^a^ (bp)	Neighboring genes	TEs	Accession Numbers
R570	085_J04	123,896	5,584	8	4	PYBL01000039.1
	096_D24	109,397	5,586	7	2	PYBL01000047.1
	104_G22	99,804	5,584	6	4	KF184821.1
	237_G04	94,577	5,589	5	3	PYBL01000103.1
SP803280	109_H09	89,338	5,470	5	1	MW166211
	149_N10	140,447	6,067	6	3	MW166210
	228_N18	141,113	5,653	8	2	MW166209
	257_A23	123,970	5,571	6	3	MW166208
	258_B07	133,749	5,585	8	4	MW166207
	264_H19	118,514	5,470	10	1	MW166206
	264_P08	132,964	5,588	12	2	MW166205
	273_J18	130,293	5,585	9	4	MW166204
	441_A04	143,827	5,468	10	5	MW166203
	453_B01	134,780	5,571	7	4	MW166202
	456_J23	126,018	5,585	8	4	MW166201
	465_H15	155,144	5,585	9	4	MW166200
	480_A02	135,325	5,588	7	4	MW166199
	492_H21	99,828	5,585	5	3	MW166198
	494_M23	135,410	5,571	8	4	MW166197
	524_N01	20,541	5,571	1	2	MW166196
	548_I17	126,419	5,588	4	5	MW166195

^a^This region comprises from the start codon of the first gene to the stop codon of the duplicate gene in tandem, including exon, intron and intergenic regions

**Fig. 2:**
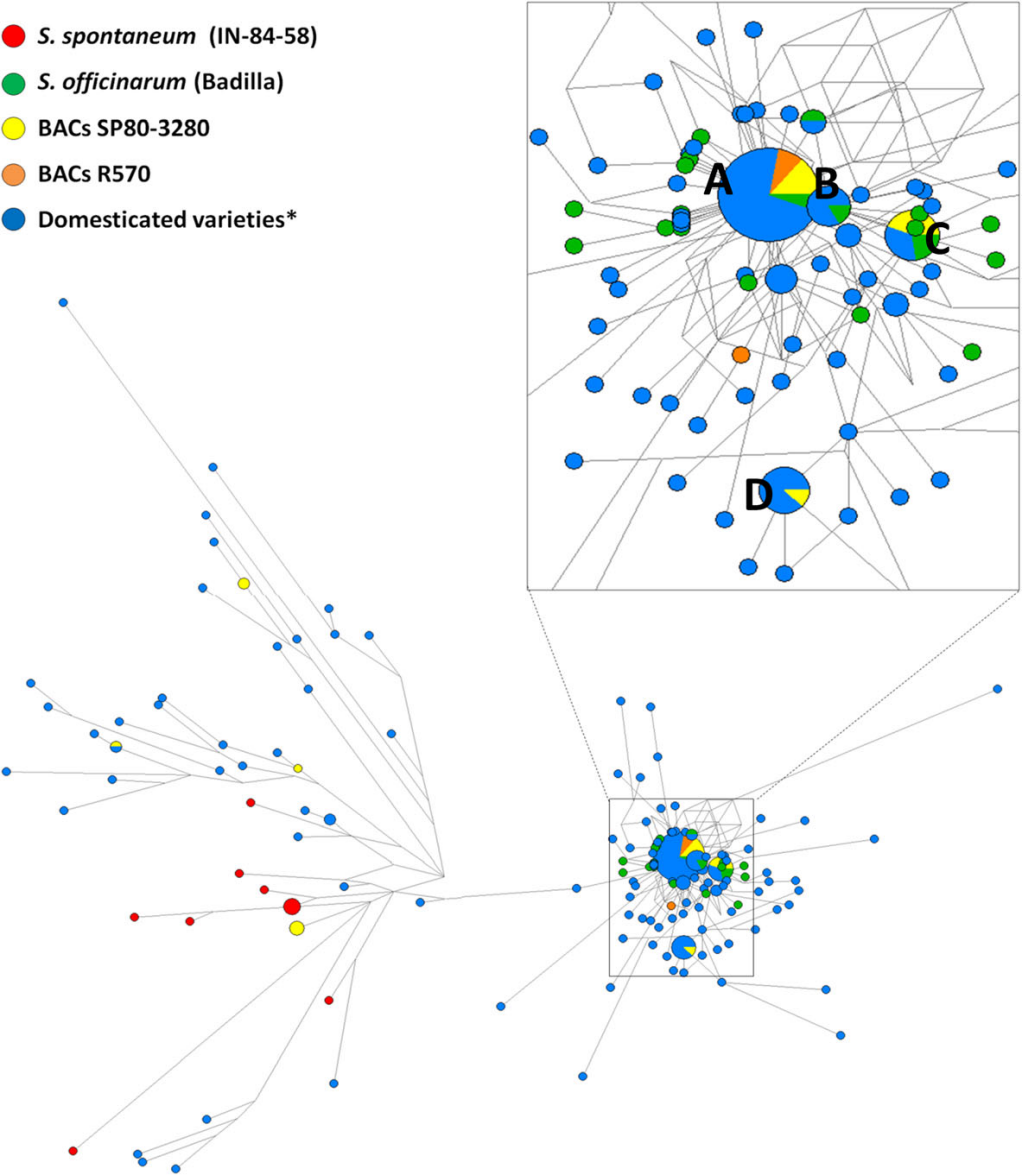
Network analysis of *S6PP* tandem duplication genomic region. The Network was built using NETWORK 5 software by Median-joining method, from the alignment of a region of approximately 1539 bp of various domesticated varieties of sugarcane, the species used as parental in the crosses (*S. spontaneum* and *S.officinarum*) and sequenced BACs of R570 and SP80–3280. The largest node was zoomed for a better visualization and is shown inside the square. **a** The largest node is formed by R570 BACs predicted to transcribe a single transcript containing two domains of *S6PP,* SP80–3280 BACs and 36 sequences from POJ-2878, NCo-310, RB-72454, NA56–79, RB-867515, SP70–1143, Miscanthus, Co-290 and RB-835486 domesticated varieties; **b** node formed by six sequences; **c** node formed by nine sequences; **d** node formed by eight sequences. The smaller nodes are formed by only one sequence. The size of the node (circle) is relative to the number of sequences in that haplotype. The distance between the nodes is proportional to the number of substitutions. (*) Refers to the domesticated varieties used in the study: Mandalay, SP70–1143, Miscanthus, Co-290, RB-835486, POJ-2878, Nco-310, RB-72454, NA 56–79, RB 867515, SP81–3250, R570, SP80–3280

NETWORK analysis allowed us to address the nucleotide diversity at population level. A fragment of 1539 nucleotides, which include the intergenic region between the two copies of *S6PP,* of all the aforementioned sequenced BACs were included in the original network alignment published by De Setta et al. [12]. Almost all the sequences grouped into four nodes, as shown in Fig. 2. All nodes are composed of domesticated varieties and SP80–3280 BACs, except node B. The R570 BACs are prevalent in the central node A, composed of 36 sequences, including *S. officinarum* (Badilla). A few *S. officinarum* sequences were clustered with SP80–3280 and hybrid cultivars used in Brazilian breeding programs (Fig. 2, node C). However, none of the *S. spontaneum* sequenced fragments were found with sugar accumulating varieties.

We investigated the promoter region of both *S6PP.1* and *S6PP.2* in our BAC collection and found that little nucleotide similarity is observed upstream the translation start-site ATG between the two genes. However, multiple nucleotide alignment within each of the promoter regions support high nucleotide identity. Analysis of the 620 nucleotides upstream of the *S6PP.1* and *S6PP.2* ATG, revealed that *S6PP.1* has a defined and conserved TATA box as well as several overlapping motifs associated to ABA and light responsiveness, a few drought and auxin responsive elements (Figure S3). All but three *S6PP.2* upstream regions do not have TATA boxes at expected positions but all share binding sites to gibberellin, auxin and light responsive elements. SP803280_480_A02, SP803280_548_H17 and SP803280_264_P08 have a TATA box in the minus strand at position 271–277.

### S6PP tandem duplication is ancestral to sugarcane

A survey in Phytozome13 database [22], revealed that the S6PP tandem duplication is not unique to sugarcane, is also present in at least two families (Poaceae and Ranunculaceae). We identified the tandem duplication in genomes of different cultivars from *Sorghum bicolor* and *Panicum hallii*, *Panicum virgatum* 5, *Setaria italica* v2.2, *Setaria viridis* v2.1, *Miscanthus sinensis* v7.1, but not in *Zea mays*, *Brachypodium distachion* nor *Oryza sativa*. The tandem duplicates are located in a fragment of ~ 5000 bases in all analyzed Poaceae, whereas in the Ranunculaceae *Aquilegia coerulea* v3.1 (highlighted in gray, Table 2) the region containing the tandem duplicates is larger, of about 8000 bp.

**Table 2:** Location of S6PP tandem duplication in Poaceae and Ranunculaceae genomes

Classification family	Genome	Location	Transcript name (Phytozome13)		Genomic tandem region^**a**^ (bp)
			***S6PP.1***	***S6PP.2***	
Ranunculaceae	*Aquilegia coerulea* v3.1	Chr 1	Aqcoe1G073100.1	Aqcoe1G073000.1	8369
Poaceae (Grasses)	*Miscanthus sinensis v7.1*	Chr 16	Misin16G055500.1	Misin16G055600.1	5129
	*Miscanthus sinensis v7.1*	Chr 17	Misin17G0551400.1	Misin17G051300.1	5283
	*Panicum hallii* v3.0	Chr 3	Pahal.3G115900.1	Pahal.3G116000.1	5581
	*Panicum halli* HAL v2.1	Chr 3	PhHAL.3G110000.1	PhHAL.3G110100.1	5590
	*Panicum virgatum* v5.1	Chr 3 N	Pavir.3NG188964.1	Pavir.3NG189313.1	6302
	*Panicum virgatum* v5.1	Chr 3 K	Pavir.3KG143200.2	Not annotated^b^	5506
	*Setaria italica* v2.2	Scaffold_3	Seita.3G059500.1	Seita.3G059600.1	5694
	*Setaria viridis* v2.1	Chr 3	Sevir.3G060400.2	Not annotated^b^	7793
	*Sorghum bicolor v3.1.1*	Chr 9	Sobic.009G040900.2	Sobic.009G041000.1	5198
	*Sorghum bicolor* Rio v2.1	Chr 9	SbRio.09G043600.1	SbRio.09G043700.1	5198
	*Sorghum bicolor_RTx430 v2.1*	Chr 9	SbiRTx430.09G042400.1	SbiRTx430.09G042500.1	7186

^a^This region comprises from the start codon of the first gene to the stop codon of the duplicate gene in tandem, including exon, intron and intergenic regions

^b^Duplication evidenced by BLAST, not annotated in Phytozome13

**Fig. 3:**
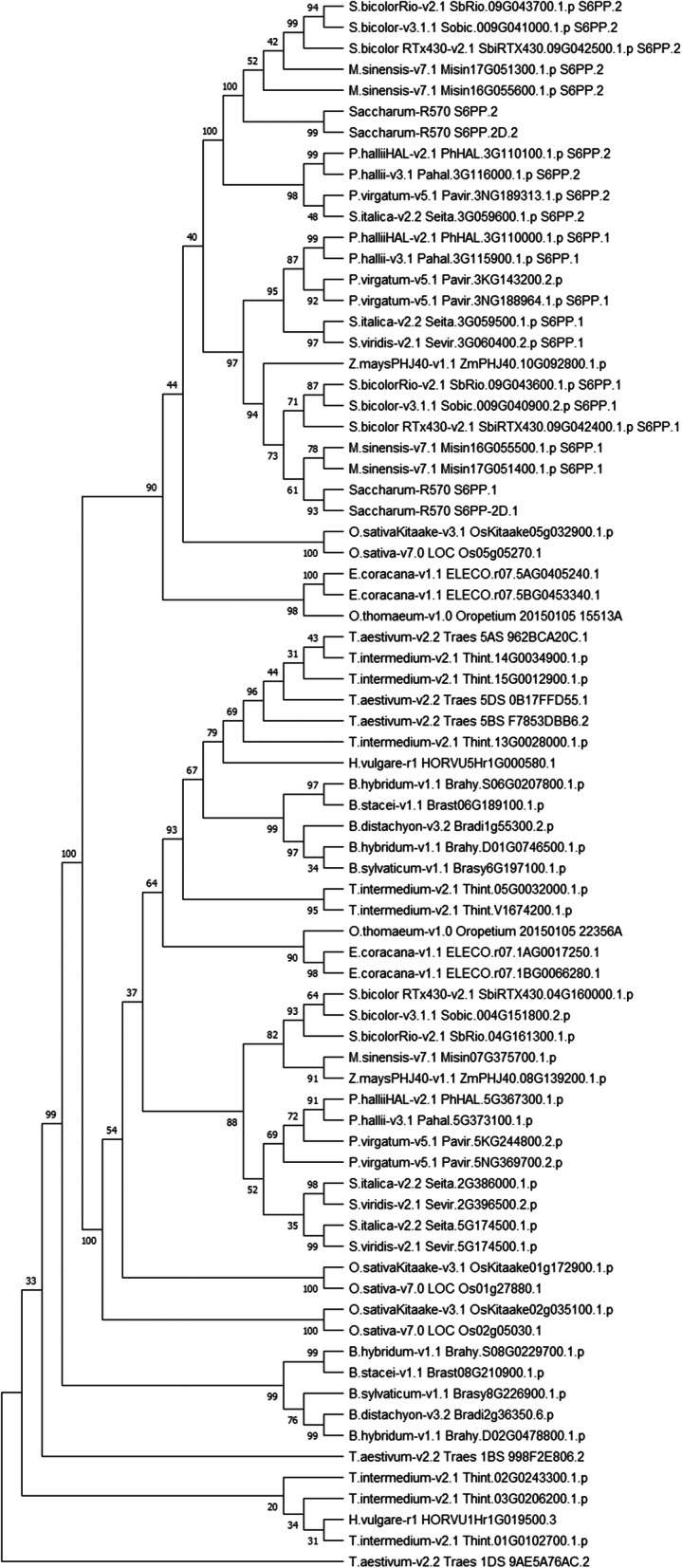
Molecular Phylogenetic tree of SPPs domains in Poaceae species. Analysis inferred by Maximum Likelihood method, JTT model, using MEGA7 software, with 1000 bootstrap pseudo-replicated. The genomes with tandem duplication are denoted by 1 or 2 when referring to each variant described in the present work

In order to determine if the duplication occurred multiple times in Poaceae or if it was ancestral, a molecular phylogenetic tree was built using only the S6PP catalytic domain (Fig. 3, Figure S4). The *S6PP_2D.1* and *S6PP_2D.2* annotation was used to explore each specific domain in the two-domain S6PP protein. Two highly supported nodes, indicate that all *S6PP.1* and all *S6PP*.2 homologs clustered together, each including the corresponding domain of *S6PP_2D*. This result supports the ancestral origin of the tandem duplication in Poaceae. Interestingly, maize and rice *S6PP* cluster with sugarcane *S6PP.1* and suggests that they may have lost the tandem duplication.

Further to investigate the selection rate of the sugarcane homologs, dN/dS calculation [23] was performed on *S6PP.1*, *S6PP.2*, *S6PP_2D*, *Miscanthus* tandem orthologs located on chromosome 17 and maize ZmSPP1 (ZmPHJ40 08G139200) and ZmSPP2 (ZmPHJ40 10G092800). Reciprocal calculations support that these genes are equally under purifying selection with similar substitution rates most probably to maintain protein function. Nonetheless, the tandemly duplicated genes are more distantly related to each other than their corresponding *Miscanthus* tandem orthologs (Table S1). When maize ZmSPP1 and ZmSPP2 are used for dN/dS calculations, the tandemly duplicated genes are more similar to ZmSPP2 in agreement with the ML tree presented in Fig. 3.

### Expression pattern of *S6PP* genes in sugar cane tissues

The expression pattern of *S6PP.1* and *S6PP_2D* in sugarcane was verified with an experiment built with following rationale: sucrose is first produced in mature leaves as a result from photosynthesis; then it is translocated through the phloem to sink tissues (apical meristem and roots). We examined the expression pattern from two commercial hybrid varieties SP80–3280 and R570, as well as two progenitor species *S. officinarum* (Badilla) and *S. spontaneum* (IN84–58) in leaves, culm, meristem and roots as well as in different developmental time points (3, 6, and 9 months).

**Fig. 4:**
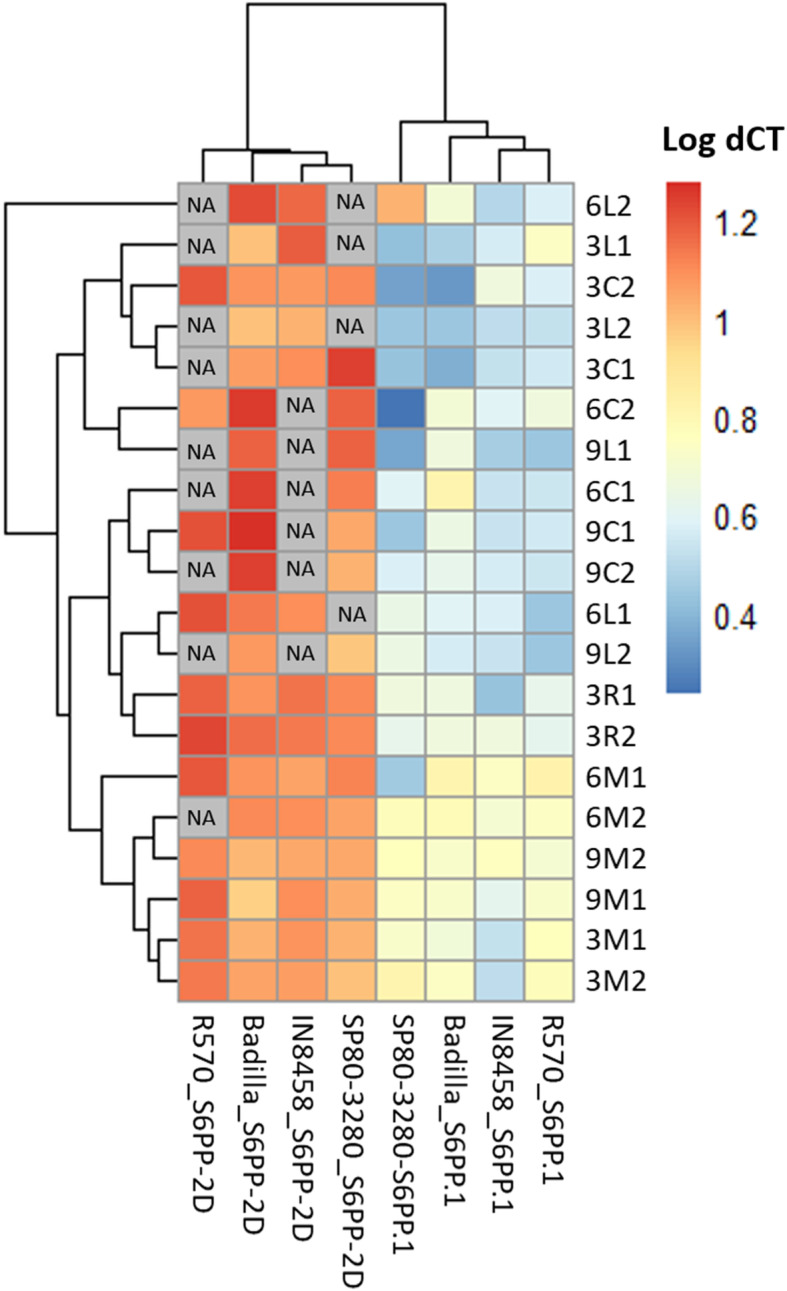
Expression profile determined by Log dCT (CT*target* - CT*housekeeping*) from RT-qPCR values in samples from sugarcane tissues; L: leaf, C: culm, M: apical meristem, R: root, in 3, 6, and 9 months old plants. The color codes indicate the ranging from blue (high relative expression) to red (low relative expression). Clusteres was determined by correlation analysis

The heat map of log delta CT values (CT value of target gene – CT value of housekeeping) [24] presented (Fig. 4) supports that the *S6PP_2D* isoform is expressed mainly at 3 months in most tissues examined, in addition to the *S6PP.1* isoform, but the expression pattern is variable. Photosynthetic tissues (leaf and culm) from commercial varieties, *S. spontaneum* and *S. officinarum* have the highest expression levels of *S6PP.1* at 3, 6 and 9 months. Differences in the expression pattern of the *S6PP.1* and *S6PP_2D* genes in sink tissues (meristem and root) is less pronounced and cluster together across plant varieties and time.

**Fig. 5:**
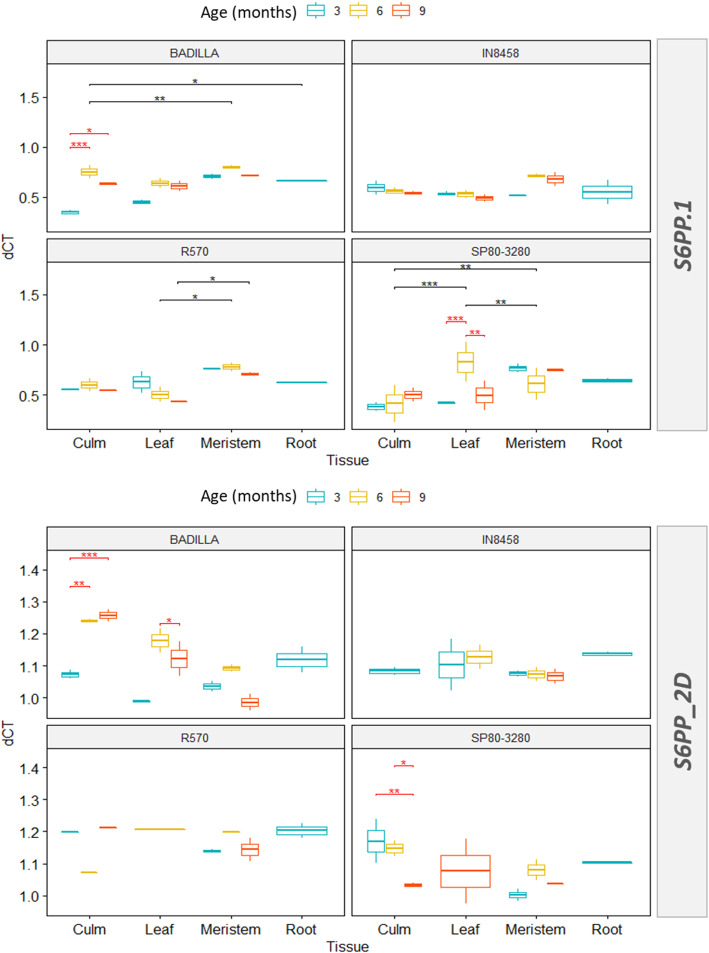
Box-plot (dCT values) of S6PP.1 and S6PP_2D transcripts profiled by RT-qPCR in samples from sugarcane cultivars (Badilla, IN8458, R570 and SP80-3280) in culm, leaf, apical meristem and root tissues, with 3, 6, 9, months age. Boxes show the median, 25th and 75th percentiles. Corrected *p*-values of < 0.05, 0.01, 0.001 are denoted by *, ** and ***, respectively. Black lines and red lines represent statistically significant differences between tissues and ages, respectively. P-adjust: Bonferroni method

A three-way ANOVA [25] analysis presented as a box plot in Fig. 5 support the interaction between cultivar, age and tissues on the expression of *S6PP.1* and *S6PP_2D* genes*.* Significant differences in the expression are observed for Badilla and SP80–3280 tissues and age whereas it is less pronounced for R570 and IN84–58. The interaction between cultivar, age and tissue is supported statistically at *p* = 0.013 for S6PP and *p* = 0.007 for *S6PP_2D.1*. Both genes in Badilla and SP80–3280 genetic background are strongly influenced by factors such as age and tissues. In addition, all simple pairwise comparisons, between the tissues and ages per cultivar, were run with a Bonferroni adjustment applied (p.adjust = 0.05) [26]. The non-accumulating sugar IN84–58 cultivar presents the least variation in the expression pattern when all conditions are compared. Conversely, the commercial SP80–3280 varies most and presents the highest expression level.

**Fig. 6:**
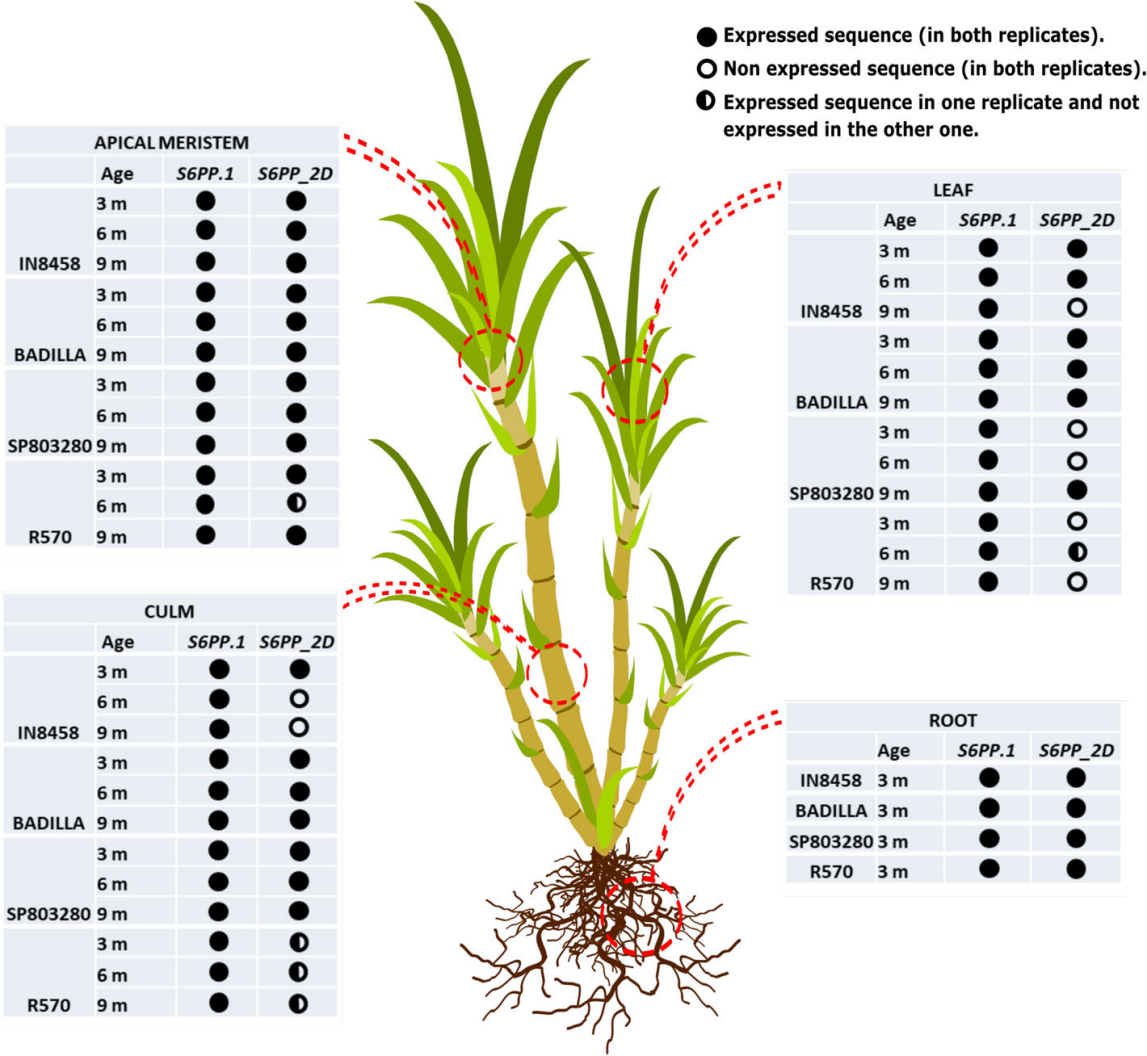
Summarized biological map of the expression of the *S6PP.1* and *S6PP_2D* genes in sugarcane cultivars, *S. spontaneum* and *S. officinarum*. Expression of *S6PP.1* and *S6PP_2D* in source (leaf and culm) and sink (root and meristem) tissues of four different plant lines are presented. Black-filled circles denote conditions in which expression was observed, white-filled circles represent absence of expression and half black- half white circles, represent that the two biological replicates analyzed are not concordant. Age is presented in months

Transcription of *S6PP* gene is expected in leaves, a photosynthesizing tissue capable of producing sucrose from the Suc6P, substrate of S6PP enzyme in the sucrose metabolic pathway. This free sucrose is translocated through the conductive vessels of the phloem for all non-photosynthetic tissues, including sink tissues, where it is accumulated after a series of interconversion reactions. The export of carbohydrates from photosynthesizing leaves (source) provides the substrate for the growth and maintenance of non-photosynthetic vegetative tissues (sink) [27] (Fig. 6). Sugars represent the main source of energy for all eukaryotic organisms, carbohydrates are essential for fundamental processes in plant growth and sugarcane tends to accumulate more sucrose near the last phase of the culture cycle, when it has a low growth rate.

According to the results presented in Fig. 4, S6PP genes are more expressed in tissues at 3 months of age and a decrease over time of *S6PP_2D* in source tissues. At early stages of the plant development when plant growth is accelerated, there is a greater requirement of sucrose [27]. Based on the expression of the *S6PP* transcripts, the results support that the gene is involved in the cyclic balance between synthesis, storage, use and re-synthesis to sustain the growth needs of the plant. Therefore, the transport and partition of sugars from phototrophic leaves (source) to heterotrophic organs (sink) through the phloem are the main parameters that control crop productivity [5].

## Discussion

Sugarcane tends to accumulate more sucrose when it has a low growth rate [28], when it reaches maturity, between 10 and 14 months [29]. Early in vitro assays suggested that the catalytic activity of S6PP enzyme is downregulated to 40% in the presence of 50 mM of sucrose in sugar cane Pindar variety [30]. Further biochemical studies reported that S6PP from sugarcane (*Saccharum* spp. cv NCO310) and red beet were inhibited 40 and 59%, respectively in the presence of 100 mM sucrose [31]. In addition, S6PP from rice (*Oryza sativa*) presented 15% inhibition in 160 mM sucrose, and S6PP from *Synechocystis* sp. PCC 6803 is inhibited in 19 and 27% with 200 mM and 660 mM sucrose, respectively [15]. It is interesting to note that under natural conditions, commercial sugarcane varieties sucrose levels can reach high concentrations up to 650 mM [32]. A comparative approach of gene expression between mature and immature culms reported by Carson et al. [33] reveal that some of the enzymes involved in the sucrose metabolism are more abundant in immature tissues whereas are less expressed in mature tissues.

The results presented here support that the S6PP expression level varies between tissues and during plant development. According to [33], the accumulation of sucrose is dependent on the size and activity of the tissue. We anticipate that the results described here support that the ability to accumulate sucrose could be related by differences of *S6PP* gene expression levels between varieties. *S. spontaneum* (variety IN-8458), in general, showed the least variation of expression of both *S6PP.1* and *S6PP_2D*, in tissues and across the developmental time analyzed but, this species is characterized by not accumulating large amounts of sucrose. However, S *spontaneum* brings increased tolerance to biotic and abiotic stresses and adaptability into breeding programs. Conversely, *S. officinarum* (Badilla), that presented the largest variation in levels of expression, is known to accumulate high levels of sucrose in the cell but has less resistance to diseases. Wang et al. [32] state that hybrid commercial varieties are capable of accumulating high concentrations of sucrose, up to 650 mM or 18% of their fresh weight at maturity. Therefore, we speculate the reduction of the *S6PP* transcripts is due to the high accumulation of sucrose.

## Conclusions

A comparative genomic approach between two commercial hybrids (R570 and SP80–3280) using sequenced BAC fragments, confirmed the presence of a tandem gene duplication in 21 genomic fragments, of which some are predicted to be transcribed as a single transcript, thus containing two S6PP domains. The phylogenetic analyses indicate that this tandem duplication is conserved in Poaceae thus, ancestral to *Saccharum*. Our RT-qPCR analyses indicate that the new isoform, *S6PP_2D,* is differentially expressed through developmental stage, depending also on the sugarcane cultivar studied. In summary, the present work describes the phylogeny and expression patterns of *S6PP* genes in sugarcane ancestral and commercial cultivars.

## Methods

### Comparative genomics

Twenty-one genomic fragments containing *S6PP* genes of the commercial hybrid sugarcane R570 and SP80–3280 were analyzed. Table 1 list the name, genomic cultivar origin, number of genes and transposable elements (TEs) identified in each genomic fragment after manual inspection and annotation. All genes predicted during BAC annotation were curated against NCBI databases and the TEs were analyzed in GIRI RePBASE [34] databases, considering 80–80-80 rule [35]. A Maximum Likelihood phylogenetic tree was carried out with MEGA7 software [36] using *T92 + G* as the best model, with 1000 bootstraps to determine the phylogeny and possible grouping and segregations between the fragments. The alignment included the tandem duplication genomic region from the start codon of the first gene sequence to the stop codon of the second tandem duplicated gene including exon, intron and intergenic regions. For comparative purposes Neighbor Joining and Parsimony phylogenetic tree were produced and no differences in grouping was observed. Aligned sequence size is indicated in the Table 1. Promoter analysis was performed by extracting 620 nucleotides upstream the *S6PP.1* and *S6PP.2* start codons, alignments were performed with MEGA7. PlantCARE [37] was used to determine the potential cis-acting regulatory elements in each region. PAL2NAL online version (http://www.bork.embl.de/pal2nal/) was used for dN/dS calculations as described (Universal Code; Removing Gaps to enable dN/dS calculations) with PALM output to build Table S1.

### Network analyses

A network analyses was carried out using a region of approximately 1539 bp of various domesticated varieties of sugarcane such as, Mandalay, SP70–1143, Miscanthus, Co-290, RB-835486, POJ-2878, Nco-310, RB-72454, NA 56–79, RB 867515, SP-81-3250, including R570, SP80–3280 and *S. spontaneum* and *S. officinarum* species, amplified by PCR from germplasm tissue collection and sequenced by de Setta et al. [12]. The sequence alignment was performed against the same region of R570 and SP80–3280 BACs by MEGA7 software, the output was imported to DnaSP 5 software [38] to calculate the number of haplotypes and distances between the analyzed sequences, using default parameters. The network graphic representation was performed with NETWORK 5 software (**NETWORK5.** Phylogenetic Network Software website: fluxus-engineering.com) using Median-joining method [39] and default parameters. Sequences are made available upon request.

### S6PP domain phylogeny in Poaceae

The phylogenetic analysis was carried out by Maximum Likelihood method, *JTT* model, with 1000 bootstrap pseudo-replicates using the S6PP catalytic domain from Poaceae genomes obtained from Phytozome13. *S6PP* gene notation variants were numbered 1 and 2 according to their position and orientation in the tandem region. *S6PP.1* and *S6PP.2* denote first gene in the genomic fragment and its tandem duplication in the genome. Evolutionary analysis was inferred in MEGAX software [40].

### Expression profile of *S6PP* genes in sugar cane tissues

#### Plant material

Cultivars used in the present work are maintained in a local germplasm collection at the botanical garden of the Departamento de Botânica – IBUSP (Sao Paulo, BR). These cultivars were kindly provided by Dr. Eugenio Ulian under a collaborative project in 2000 and since kept under vegetative propagation. The expression profile of the *S6PP* genes in the isoforms *S6PP.1* and *S6PP_2D* was investigated, in commercial cultivars R570 and SP803280, and in the species *Saccharum officinarum* (Badilla) and *S. spontaneum* (IN84–58). Plants were grown for 12 months and plant tissue (leaf, culm, apical meristem and root) sampled at three, six, nine and 12 months from 2018 to 2019 growing season. Lateral buds were initially planted on vegetable substrate and vermiculite, supplemented with phosphate and fertilizer, at the IBUSP greenhouse with natural light and normal environmental conditions. At 3–4 weeks after bud development, plants were transferred to large pots according to plant size. Each sample was grinded to a fine powder using TissueLyzer II (QIAGEN®) and kept at − 80 °C until used.

#### RNA extraction and cDNA synthesis

Total RNA was isolate employing TRIzol® reagent (Invitrogen®), following manufacturer instructions, and digested with DNAse TURBO DNA-free (Ambion®, Life Technologies™) to remove any contaminating genomic DNA. Total RNA was visualized by agarose gel, to determine integrity. Total RNA was used as cDNA template by using reverse transcriptase *SuperScript™ III* (Invitrogen®). cDNA samples were kept at − 20 °C until used.

### Expression quantification by qPCR

In order to quantify the expression of *S6PP.1* and *S6PP_2D* by qPCR, specific primers were designed on CDS predicted sequences. The primer design involved an exon-exon junction to reduce the risk of false positives by any contaminating genomic DNA, with an amplicon size of 100 bp. Primers s6pp.1-2D_F (5′-*CTC AGC CAG AGA GGA ATC AG-3′*) and s6pp.1-2D_R (5′-*CAC GTT TCT CCA ACT TCT GTG*-3′) were used to amplify both *S6PP.1* and *S6PP_2D.* Whereas primers s6pp-2D_F (5′-*ACA CGT TCA TCT TGG AAC CC-3′*) and s6pp-2D_R (5′-*ATC ATA AGA CGG GCT GAA GC*-3′) were used to characterize expression of the isoform *S6PP_2D* in the region that was exonized.

The experiment was normalized with the housekeeping gene eukaryotic initiation factor 4-alpha (*eIF-4α*), amplified with primers eIF4a_F (5′*- TTG TGC TGG ATG AAG CTG ATG −* 3′) and eIF4a_R (5′- *GGA AGA AGC TGG AAG ATA TCA TAG A -3′)* [41]*.* Quantitative PCR (qPCR) was performed with SsoAdvanced™ Universal SYBR® Green Supermix kit (*BIO-RAD)* following the manufacturer’s instructions.

Expression data was obtained from two independent biological replicates each with three technical replicates in a QuantStudio 7 Flex Real-Time PCR (Applied Biosystems) comprised 2 min denaturation at 50 °C, 30 s at 98 °C and then followed by 40 cycles (98 °C for 15 s and 60 °C for 60 s). To check the specificity of the amplicon, the qRT-PCR products of each gene were used for analysis of melting curves in each reaction (data not shown).

### Data analysis

The dCT values were obtained across difference of CT values between the target genes and housekeeping (*CT target* – *CT housekeeping*) for each sample. A heat map was made for these data, the values were plotted logarithmic scale (Log dCT) [24] and clusterized by correlation method. A three-way ANOVA [25] was conducted to determine the effects of cultivar, age and tissues factors on expression genes *S6PP.1* and *S6PP_2D*. Residual analysis was performed to test for the assumptions of the three-way ANOVA. Normality was assessed using Shapiro-Wilk’s normality test.

## Supplementary Information

**Additional file 1: Supplementary Figure 1.** Schematic representation of *S6PP* and *S6PP_2D* genes. (A) Representation of S6PP.1 and S6PP.2 gene structure as identified in the genomic fragment of the BAC SCHRBa_237_G04. Dark and light blue boxes represent Exons while black arrows Introns. (B) Representation of S6PP_2D gene structure as identified in the genomic fragment of the BAC SCHRBa_104_G22. Dark and light blue boxes represent Exons while black arrows Introns. The red box corresponds to the exonized region not found in the single domain isoforms.

**Additional file 2: Supplementary Figure 2**. Graphic representation of the genomic fragments BACs aligned by the S6PP genes**.** All genes predicted during BAC annotation were curated against NCBI databases and the TEs were analyzed in GIRI RePBASE® databases, considering 80–80-80 rule (Wicker et al. 2007) and are shown in the figure. Black rectangles indicate BACs of the variety SP80–3280; purple rectangles represent the BACs of the R570 variety, red rectangles represent the *S6PP* genes, green rectangles represent neighboring genes of another nature, the blank arrows within the rectangles represent the direction of transcription, blue rectangles represent transposing elements, the shading between the rectangles indicates that it is the same gene or transposing element.

**Additional file 3: Supplementary Figure 3**. Multiple alignment of S6PP.1 upstream region.

**Additional file 4: Supplementary Figure 4.** Poaceae S6PP domain multiple alignement.

**Additional file 5: Supplmentary Figure 5.** S6PP dNdS from sugarcane and close relatives.

## Data Availability

The datasets generated and/or analyzed during the current study are available in the GenBank repository under the accession numbers PYBL01000039.1, PYBL01000047.1, PYBL01000103.1, KF184821.1, MW166195, MW166196, MW166197, MW166198, MW166199, MW166200, MW166201, MW166202, MW166203, MW166204, MW166205, MW166206, MW166207, MW166208, MW166209, MW166210, and MW166211. The datasets used and/or analyzed during the current study are available from the corresponding author on reasonable request.

## Abbreviations

S6PP: Sucrose-phosphate phosphatase
SPS: Sucrose phosphate synthase
SuSy: Sucrose synthase
BAC: Bacterial Artificial Chromosomes
TE: Transposable elements
CDS: Coding sequence
