# Supplementary material for: Sucrose-phosphate phosphatase from sugarcane reveals an ancestral tandem duplication
Source: BMC Plant Biol. 2021 Jan 7;21:23. doi: 10.1186/s12870-020-02795-5 (PMC7792115; doi:10.1186/s12870-020-02795-5)
Supplement: Supplementary file 1 — Additional file 1: Supplementary Figure 1. Schematic representation of S6PP and S6PP_2D genes. (A) Representation of S6PP.1 and S6PP.2 gene structure as identified in the genomic fragment of the BAC SCHRBa_237_G04. Dark and light blue boxes represent Exons while black arrows Introns. (B) Representation of S6PP_2D gene structure as identified in the genomic fragment of the BAC SCHRBa_104_G22. Dark and light blue boxes represent Exons while black arrows Introns. The red box corresponds to the exonized region not found in the single domain isoforms. [file 12870_2020_2795_MOESM1_ESM.pdf]

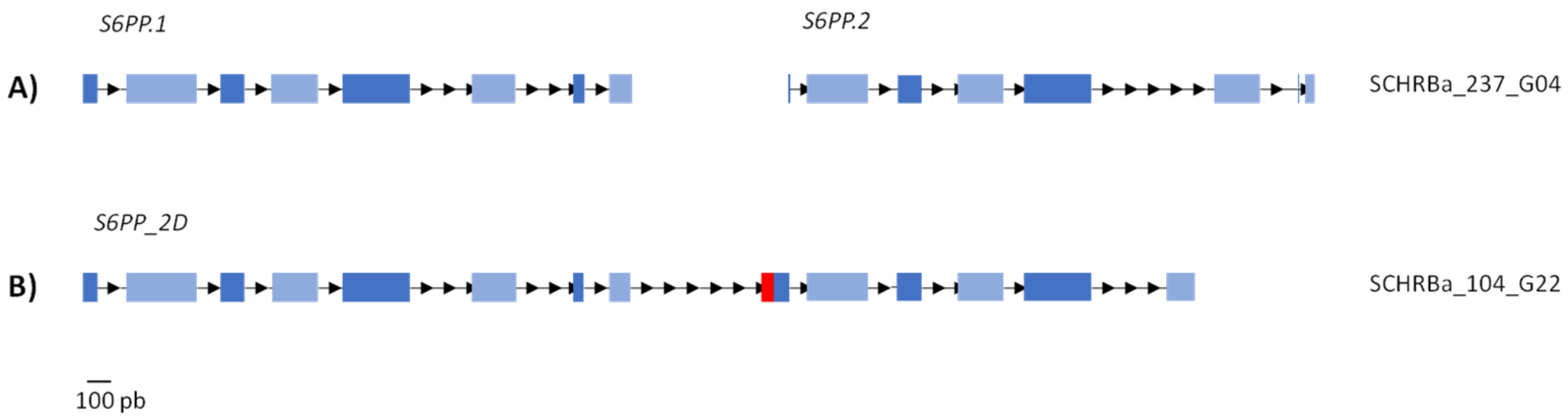

Supplementary Figure 1. Schematic representation of *S6PP* and *S6PP_2D* genes. (A) Representation of S6PP.1 and S6PP.2 gene structure as identified in the genomic fragment of the BAC SCHRBa_237_G04. Dark and light blue boxes represent Exons while black arrows Introns. (B) Representation of S6PP_2D gene structure as identified in the genomic fragment of the BAC SCHRBa_104_G22. Dark and light blue boxes represent Exons while black arrows Introns. The red box corresponds to the exonized region not found in the single domain isoforms.
