# Supplementary material for: Sucrose-phosphate phosphatase from sugarcane reveals an ancestral tandem duplication
Source: BMC Plant Biol. 2021 Jan 7;21:23. doi: 10.1186/s12870-020-02795-5 (PMC7792115; doi:10.1186/s12870-020-02795-5)
Supplement: Supplementary file 2 — Additional file 2: Supplementary Figure 2. Graphic representation of the genomic fragments BACs aligned by the S6PP genes. All genes predicted during BAC annotation were curated against NCBI databases and the TEs were analyzed in GIRI RePBASE® databases, considering 80–80-80 rule (Wicker et al. 2007) and are shown in the figure. Black rectangles indicate BACs of the variety SP80–3280; purple rectangles represent the BACs of the R570 variety, red rectangles represent the S6PP genes, green rectangles represent neighboring genes of another nature, the blank arrows within the rectangles represent the direction of transcription, blue rectangles represent transposing elements, the shading between the rectangles indicates that it is the same gene or transposing element. [file 12870_2020_2795_MOESM2_ESM.pdf]

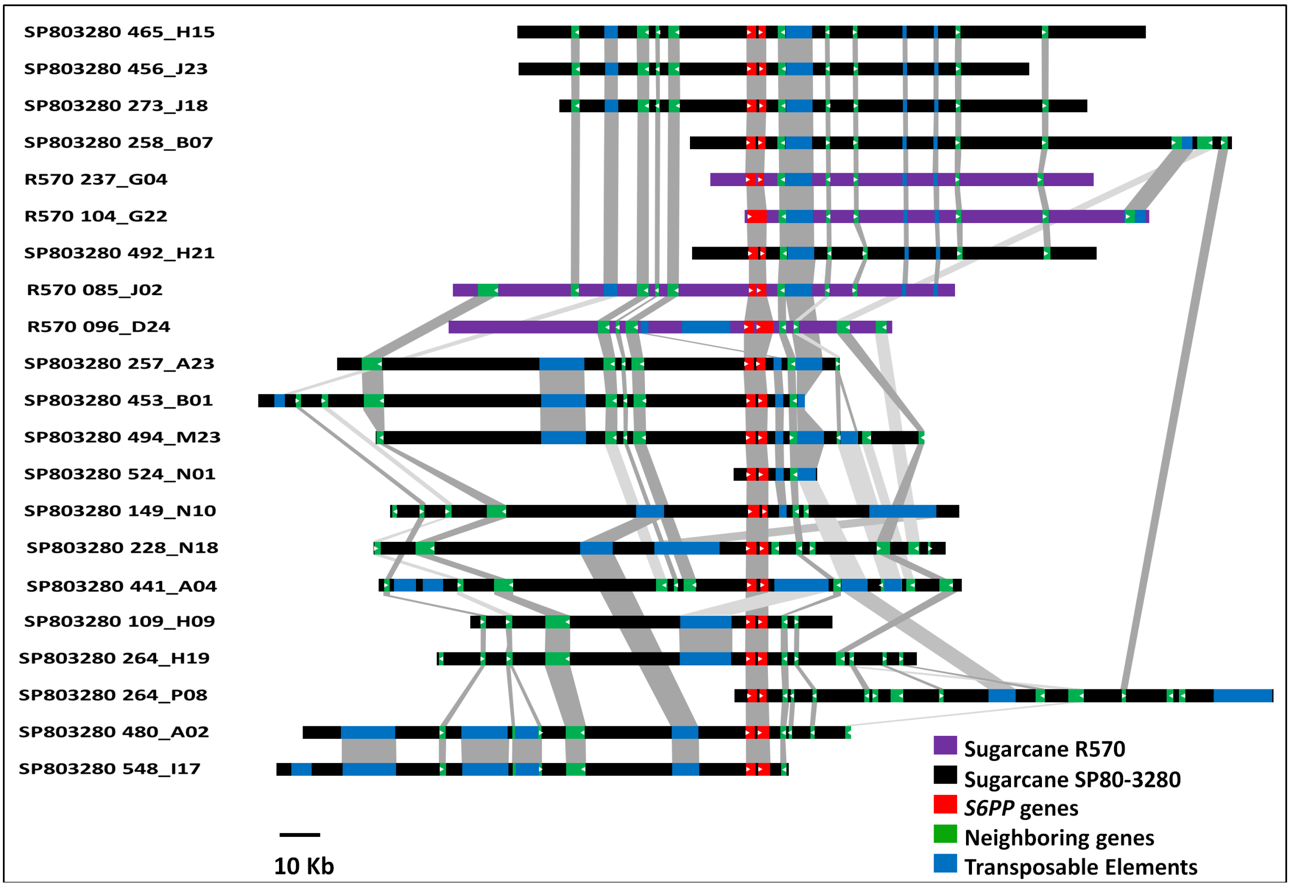

Supplementary Figure 2. Graphic representation of the genomic fragments BACs aligned by the S6PP genes. All genes predicted during BAC annotation were curated against NCBI databases and the TEs were analyzed in GIRI RePBASE® databases, considering 80-80-80 rule (Wicker et al. 2007) and are shown in the figure. Black rectangles indicate BACs of the variety SP80-3280; purple rectangles represent the BACs of the R570 variety, red rectangles represent the *S6PP* genes, green rectangles represent neighboring genes of another nature, the blank arrows within the rectangles represent the direction of transcription, blue rectangles represent transposing elements, the shading between the rectangles indicates that it is the same gene or transposing element.
