# Supplementary material for: Sucrose-phosphate phosphatase from sugarcane reveals an ancestral tandem duplication
Source: BMC Plant Biol. 2021 Jan 7;21:23. doi: 10.1186/s12870-020-02795-5 (PMC7792115; doi:10.1186/s12870-020-02795-5)
Supplement: Supplementary file 3 — Additional file 3: Supplementary Figure 3. Multiple alignment of S6PP.1 upstream region. [file 12870_2020_2795_MOESM3_ESM.pdf]

Supplementary figure 3: Multiple Alignment of S6PP.1 upstream region.

```
                   1
R570_104_G22       CGCAGCCATT AGATATTTAG ATGGCGATCT TGCTATTTAC TAGCATCTCA CATGGGGTGG TATA------ GGTGTGGGTG
R570_237_G04       CGCAGCCATT AGATATTTAG ATGGCGATCT TGCTATTTAC TAGCATCTCA CATGGGGTGG TATA------ GGTGTGGGTG
R570_096_D24       CTCAGCCATT AGATATTTAG ATGGCGATCT TGCTATTTAC TAGCATCTCA CATGGGGTGG TATA------ ------GGTG
R570_085_J02       CGCAGCCATT AGATATTTAG ATGGCGATCT TGCTATTTAC TAGCATCTCA CATGGGGTGG TATA------ GGTGTGGGTG
SP803280_264_P08   CTCAGCCATT AGATATTTAG ATGGCGATCT TGCTCTTTAC TAGCATCTCA CATGGGGTGG TATA------ ------GGTG
SP803280_480_A02   CTCAGCCATT AGATATTTAG ATGGCGATCT TGCTCTTTAC TAGCATCTCA CATGGGGTGG TATA------ ------GGTG
SP803280_548_I17   CTCAGCCATT AGATATTTAG ATGGCGATCT TGCTCTTTAC TAGCATCTCA CATGGGGTGG TATA------ ------GGTG
SP803280_109_H09   CTCAGCCATT AGATATTTAG ATGGCGATCT TGCTATTTAC TAGCATCTCA CATGGGGTGG TATA------ GGTGTGGGTG
SP803280_264_H19   CTCAGCCATT AGATATTTAG ATGGCGATCT TGCTATTTAC TAGCATCTCA CATGGGGTGG TATA------ GGTGTGGGTG
SP803280_228_N18   CGCAGCCATT AGATATTTAG ATGGCGATCT TGCTATTTAC TAGCATCTCA CATGGGGTGG TATA------ GGTGTGGGTG
SP803280_441_A04   CTCAGCCATT AGATATTTAG ATGGCGATCT TGCTATTTAC TAGCATCTCA CATGGGGTTG TATA------ GGTGTGGGTG
SP803280_258_B07   CGCAGCCATT AGATATTTAG ATGGCGATCT TGCTATTTAC TAGCATCTCA CATGGGGTGG TATA------ GGTGTGGGTG
SP803280_273_J18   CGCAGCCATT AGATATTTAG ATGGCGATCT TGCTATTTAC TAGCATCTCA CATGGGGTGG TATA------ GGTGTGGGTG
SP803280_465_H15   CGCAGCCATT AGATATTTAG ATGGCGATCT TGCTATTTAC TAGCATCTCA CATGGGGTGG TATA------ GGTGTGGGTG
SP803280_456_J23   CGCAGCCATT AGATATTTAG ATGGCGATCT TGCTATTTAC TAGCATCTCA CATGGGGTGG TATA------ GGTGTGGGTG
SP803280_492_H21   CGCAGCCATT AGATATTTAG ATGGCGATCT TGCTATTTAC TAGCATCTCA CATGGGGTGG TATA------ GGTGTGGGTG
SP803280_149_N10   CTCAGCCATT AGATATTTAG ATGGCGATCT TGCTATTTAC TAGCATCTCA CATGGGGTTG TATAGGTGTG GGTGTGGGTG
SP803280_257_A23   CTCAGCCATT AGATATTTAG ATGGCGATCT TGCTATTTAC TAGCATCTCA CATGGGGTTG TATA------ GGTGTGGGTG
SP803280_453_B01   CTCAGCCATT AGATATTTAG ATGGCGATCT TGCTATTTAC TAGCATCTCA CATGGGGTTG TATA------ GGTGTGGGTG
SP803280_494_M23   CTCAGCCATT AGATATTTAG ATGGCGATCT TGCTATTTAC TAGCATCTCA CATGGGGTTG TATA------ GGTGTGGGTG
SP803280_524_N01   CTCAGCCATT AGATATTTAG ATGGCGATCT TGCTATTTAC TAGCATCTCA CATGGGGTTG TATA------ GGTGTGGGTG

                  81
R570_104_G22       TGGGTGTGGG GATAGTCGCT TAGCAGCCGG AAAGGCGGAA ACGTGAAACA TGGCCACGTT CCTTGTTCTG CCAACGTCGT
R570_237_G04       TGGGTGTGGG GATAGTCGCT TAGCAGCCGG AAAGGCGGAA ACGTGAAACA TGGCCACGTT CCTTGTTCTG CCAACGTCGT
R570_096_D24       TGGGTGTGGG GATAGTCGCT TAGCAGCCGG AAAGGCGGAA ACGTGAAACA TGGCCACGTT CCTTGTTCTG CCAACGTCGT
R570_085_J02       TGGGTGTGGG GATAGTCGCT TAGCAGCCGG AAAGGCGGAA ACGTGAAACA TGGCCACGTT CCTTGTTCTG CCAACGTCGT
SP803280_264_P08   TGGGTGTGGG GATAGTTGCT TAGCAGCCGG AAAGGCGGAA ACGTGAAACA TGGCCA-GTT CCTTGTTCTG CCAACGTCGT
SP803280_480_A02   TGGGTGTGGG GATAGTTGCT TAGCAGCCGG AAAGGCGGAA ACGTGAAACA TGGCCA-GTT CCTTGTTCTG CCAACGTCGT
SP803280_548_I17   TGGGTGTGGG GATAGTTGCT TAGCAGCCGG AAAGGCGGAA ACGTGAAACA TGGCCA-GTT CCTTGTTCTG CCAACGTCGT
SP803280_109_H09   TGGGTGTGGG GATAGTTGCT TAGCAGCCGG AAAGGCGGAA ACGTGAAACA TGGCCA-GTT CCTTGTTCTG CCAACGTCGT
SP803280_264_H19   TGGGTGTGGG GATAGTTGCT TAGCAGCCGG AAAGGCGGAA ACGTGAAACA TGGCCA-GTT CCTTGTTCTG CCAACGTCGT
SP803280_228_N18   TGGGTGTGGG GATAGTCGCT TAGCAGCCGG AAAGGCGGAA ACGTGAAACA TGGCCA-GTT CCTTGTTCTG CCAACGTCGT
SP803280_441_A04   TGGGTGTGGG GATAGTTGCT TAGCAGCCGG AAAGGCGGAA ACGTGAAACA TGGCCA-GTT CCTTGTTCTG CCAACGTCGT
SP803280_258_B07   TGGGTGTGGG GATAGTCGCT TAGCAGCCGG AAAGGCGGAA ACGTGAAACA TGGCCACGTT CCTTGTTCTG CCAACGTCGT
SP803280_273_J18   TGGGTGTGGG GATAGTCGCT TAGCAGCCGG AAAGGCGGAA ACGTGAAACA TGGCCACGTT CCTTGTTCTG CCAACGTCGT
SP803280_465_H15   TGGGTGTGGG GATAGTCGCT TAGCAGCCGG AAAGGCGGAA ACGTGAAACA TGGCCACGTT CCTTGTTCTG CCAACGTCGT
SP803280_456_J23   TGGGTGTGGG GATAGTCGCT TAGCAGCCGG AAAGGCGGAA ACGTGAAACA TGGCCACGTT CCTTGTTCTG CCAACGTCGT
SP803280_492_H21   TGGGTGTGGG GATAGTCGCT TAGCAGCCGG AAAGGCGGAA ACGTGAAACA TGGCCACGTT CCTTGTTCTG CCAACGTCGT
SP803280_149_N10   TGGGTGTGGG GATAGTTGCT TAGCAGCCGG AAAGGCGGAA ACGTGAAACA TGGCCA-GTT CCTTGTTCTG CCAACGTCGT
SP803280_257_A23   TGGGTGTGGG GATAGTTGCT TAGCAGCCGG AAAGGCGGAA ACGTGAAACA TGGCCA-GTT CCTTGTTCTG CCAACGTCGT
SP803280_453_B01   TGGGTGTGGG GATAGTTGCT TAGCAGCCGG AAAGGCGGAA ACGTGAAACA TGGCCA-GTT CCTTGTTCTG CCAACGTCGT
SP803280_494_M23   TGGGTGTGGG GATAGTTGCT TAGCAGCCGG AAAGGCGGAA ACGTGAAACA TGGCCA-GTT CCTTGTTCTG CCAACGTCGT
SP803280_524_N01   TGGGTGTGGG GATAGTTGCT TAGCAGCCGG AAAGGCGGAA ACGTGAAACA TGGCCA-GTT CCTTGTTCTG CCAACGTCGT

                 161
R570_104_G22       TCTAGGAACA TTCACG-TTT CCATTCCGAG AACATTTATG TTGCCATAGT ACAAAAACAT TATAAAGTAT CGCTCCATGT
R570_237_G04       TCTAGGAACA TTCACG-TTT CCATTCCGAG AACATTTATG TTGCCATAGT ACAAAAACAT TATAAAGTAT CGCTCCATGT
R570_096_D24       TCTAGGAACA TTCACG-TTT CCATTCCGAG AACATTTATG TTGCCATAGT ACAAAAACAT TATAAAGTAT CGCTCCATGT
R570_085_J02       TCTAGGAACA TTCACG-TTT CCATTCCGAG AACATTTATG TTGCCATAGT ACAAAAACAT TATAAAGTAT CGCTCCATGT
SP803280_264_P08   TCTAGGAACA TTCACG-TTT CCATTCCGAG AGCATTTATG TTGCCATAGT ACAAAAACAT TATAAAGTAT CGCTCCATGT
SP803280_480_A02   TCTAGGAACA TTCACG-TTT CCATTCCGAG AGCATTTATG TTGCCATAGT ACAAAAACAT TATAAAGTAT CGCTCCATGT
SP803280_548_I17   TCTAGGAACA TTCACG-TTT CCATTCCGAG AGCATTTATG TTGCCATAGT ACAAAAACAT TATAAAGTAT CGCTCCATGT
SP803280_109_H09   TCTAGGAACA TTCACGTTTT CCATTCTGAG AACATTTATG TTGCCATAGT ACAAAAACAT TATGAAGTAT CGCTCCATGT
SP803280_264_H19   TCTAGGAACA TTCACGTTTT CCATTCTGAG AACATTTATG TTGCCATAGT ACAAAAACAT TATGAAGTAT CGCTCCATGT
SP803280_228_N18   TCTAGGAACA TTCACG-TTT CCATTCTGAG AACATTTATG TTGCCATAGT ACAAAAACAT TATAAAGTAT CGCTCCATGT
SP803280_441_A04   TCTAGGAACA TTCACG-TTT CCATTCTGAG AACATTTATG TTGCCATAGT ACAAAAACAT TATAAAGTAT CGCTCCATGT
SP803280_258_B07   TCTAGGAACA TTCACG-TTT CCATTCCGAG AACATTTATG TTGCCATAGT ACAAAAACAT TATAAAGTAT CGCTCCATGT
SP803280_273_J18   TCTAGGAACA TTCACG-TTT CCATTCCGAG AACATTTATG TTGCCATAGT ACAAAAACAT TATAAAGTAT CGCTCCATGT
SP803280_465_H15   TCTAGGAACA TTCACG-TTT CCATTCCGAG AACATTTATG TTGCCATAGT ACAAAAACAT TATAAAGTAT CGCTCCATGT
SP803280_456_J23   TCTAGGAACA TTCACG-TTT CCATTCCGAG AACATTTATG TTGCCATAGT ACAAAAACAT TATAAAGTAT CGCTCCATGT
SP803280_492_H21   TCTAGGAACA TTCACG-TTT CCATTCCGAG AACATTTATG TTGCCATAGT ACAAAAACAT TATAAAGTAT CGCTCCATGT
SP803280_149_N10   TCTAGGAACA TTCACG-TTT CCATTCTGAG AACATTTATG TTGCCATAGT ACAAAAACAT TATAAAGTAT CGCTCCATGT
SP803280_257_A23   TCTAGGAACA TTCACG-TTT CCATTCTGAG AACATTTATG TTGCCATAGT ACAAAAACAT TATAAAGTAT CGCTCCATGT
SP803280_453_B01   TCTAGGAACA TTCACG-TTT CCATTCTGAG AACATTTATG TTGCCATAGT ACAAAAACAT TATAAAGTAT CGCTCCATGT
SP803280_494_M23   TCTAGGAACA TTCACG-TTT CCATTCTGAG AACATTTATG TTGCCATAGT ACAAAAACAT TATAAAGTAT CGCTCCATGT
SP803280_524_N01   TCTAGGAACA TTCACG-TTT CCATTCTGAG AACATTTATG TTGCCATAGT ACAAAAACAT TATAAAGTAT CGCTCCATGT

                 241
R570_104_G22       TCC-CGGTCC CGTTCCC--T TCCGTGAACA TGGCTGCTAT GGATAGATGC ATAGAGTTTC CTATATGCTC CACATATACA
R570_237_G04       TCC-CGGTCC CGTTCCC--T TCCGTGAACA TGGCTGCTAT GGATAGATGC ATAGAGTTTC CTATATGCTC CACATATACA
R570_096_D24       TCC-CGGTCC CGTTCCC--T TCCGTGAACA TGGCTGCTAT GGATAGATGC ATAGAGTTTC CTATATGCTC CACATATACA
R570_085_J02       TCC-CGGTCC CGTTCCC--T TCCGTGAACA TGGCTGCTAT GGATAGATGC ATAGAGTTTC CTATATGCTC CACATATACA
SP803280_264_P08   TCC-CGGTCC CATTCCACGT TCCGTGAACA TGGCTGCTAT GGATAGATGC ATAGAGTTTC CTATATGCTC CACATATACA
SP803280_480_A02   TCC-CGGTCC CATTCCACGT TCCGTGAACA TGGCTGCTAT GGATAGATGC ATAGAGTTTC CTATATGCTC CACATATACA
SP803280_548_I17   TCC-CGGTCC CATTCCACGT TCCGTGAACA TGGCTGCTAT GGATAGATGC ATAGAGTTTC CTATATGCTC CACATATACA
SP803280_109_H09   TCCACGTTCC CGTTCCC--T TCCGTGAACA TGGCTGCTAT GGATAGATGC ATAGAGTTTC CTATATGCTC CACATATACA
SP803280_264_H19   TCCACGTTCC CGTTCCC--T TCCGTGAACA TGGCTGCTAT GGATAGATGC ATAGAGTTTC CTATATGCTC CACATATACA
SP803280_228_N18   TCC-CGGTCC CGTTCCC--T TCCGTGAACA TGGCTGCTAT GGATAGATGC ATAGAGTTTC CTATATGCTC CACATATACA
SP803280_441_A04   TCC-CGGTCC CGTTCCC--T TCCGTGAACA TGGCTGCTAT GGATAGATGC ATAGAGTTTC CTATATGCTC CACATATACA
SP803280_258_B07   TCC-CGGTCC CGTTCCC--T TCCGTGAACA TGGCTGCTAT GGATAGATGC ATAGAGTTTC CTATATGCTC CACATATACA
SP803280_273_J18   TCC-CGGTCC CGTTCCC--T TCCGTGAACA TGGCTGCTAT GGATAGATGC ATAGAGTTTC CTATATGCTC CACATATACA
SP803280_465_H15   TCC-CGGTCC CGTTCCC--T TCCGTGAACA TGGCTGCTAT GGATAGATGC ATAGAGTTTC CTATATGCTC CACATATACA
SP803280_456_J23   TCC-CGGTCC CGTTCCC--T TCCGTGAACA TGGCTGCTAT GGATAGATGC ATAGAGTTTC CTATATGCTC CACATATACA
SP803280_492_H21   TCC-CGGTCC CGTTCCC--T TCCGTGAACA TGGCTGCTAT GGATAGATGC ATAGAGTTTC CTATATGCTC CACATATACA
SP803280_149_N10   TCC-CGGTCC CGTTCCC--T TCCGTGAACA TGGCTGCTAT GGATAGATGC ATAGAGTTTC CTATATGCTC CACATATACA
SP803280_257_A23   TCC-CGGTCC CGTTCCC--T TCCGTGAACA TGGCTGCTAT GGATAGATGC ATAGAGTTTC CTATATGCTC CACATATACA
SP803280_453_B01   TCC-CGGTCC CGTTCCC--T TCCGTGAACA TGGCTGCTAT GGATAGATGC ATAGAGTTTC CTATATGCTC CACATATACA
SP803280_494_M23   TCC-CGGTCC CGTTCCC--T TCCGTGAACA TGGCTGCTAT GGATAGATGC ATAGAGTTTC CTATATGCTC CACATATACA
SP803280_524_N01   TCC-CGGTCC CGTTCCC--T TCCGTGAACA TGGCTGCTAT GGATAGATGC ATAGAGTTTC CTATATGCTC CACATATACA
```

```
                     321
R570_104_G22         TACGCAGCAG TCAGCAATGC TTATCTCCTC GCCTTCCTTA TCATCAGTAG CTAGGACAGT AGCTTGTGTG GATGG-----
R570_237_G04         TACGCAGCAG TCAGCAATGC TTATCTCCTC GCCTTCCTTA TCATCAGTAG CTAGGACAGT AGCTTGTGTG GATGG-----
R570_096_D24         TACGCAGCAG TCAGCAATGC TTATCTCCTC GCCTTCCTTA TCATCTGTAG CTAGGACAGT AGCTTGTGTG GATGG-----
R570_085_J02         TACGCAGCAG TCAGCAATGC TTATCTCCTC GCCTTCCTTA TCATCAGTAG CTAGGACAGT AGCTTGTGTG GATGG-----
SP803280_264_P08     TACGCAGCAG TCAGCAATGC TTATCTCCTC GCCTTCCTTA TCATCAGTAG CTAGGACAGT AGCTTGTGTG GATGG-----
SP803280_480_A02     TACGCAGCAG TCAGCAATGC TTATCTCCTC GCCTTCCTTA TCATCAGTAG CTAGGACAGT AGCTTGTGTG GATGG-----
SP803280_548_I17     TACGCAGCAG TCAGCAATGC TTATCTCCTC GCCTTCCTTA TCATCAGTAG CTAGGACAGT AGCTTGTGTG GATGG-----
SP803280_109_H09     TACGCAGCAG TCAGCAATGC TTATCTCCTC GCCTTCCTTA TCATCAGTAG CTAGGACAGT AGCTTGTGTG GATGG-----
SP803280_264_H19     TACGCAGCAG TCAGCAATGC TTATCTCCTC GCCTTCCTTA TCATCAGTAG CTAGGACAGT AGCTTGTGTG GATGG-----
SP803280_228_N18     TACGCAGCAG TCAGCAATGC TTATCTCCTC GCCTTCCTTA TCATCAGTAG CTAGGACAGT AGCTTGTGTG GATGG-----
SP803280_441_A04     TACGCAGCAG TCAGCAATGC TTATCTCCTC GCCTTCCTTA TCATCAGTAG CTAGGACAGT AGCTTGCGTG GATGG-----
SP803280_258_B07     TACGCAGCAG TCAGCAATGC TTATCTCCTC GCCTTCCTTA TCATCAGTAG CTAGGACAGT AGCTTGTGTG GATGG-----
SP803280_273_J18     TACGCAGCAG TCAGCAATGC TTATCTCCTC GCCTTCCTTA TCATCAGTAG CTAGGACAGT AGCTTGTGTG GATGG-----
SP803280_465_H15     TACGCAGCAG TCAGCAATGC TTATCTCCTC GCCTTCCTTA TCATCAGTAG CTAGGACAGT AGCTTGTGTG GATGG-----
SP803280_456_J23     TACGCAGCAG TCAGCAATGC TTATCTCCTC GCCTTCCTTA TCATCAGTAG CTAGGACAGT AGCTTGTGTG GATGG-----
SP803280_492_H21     TACGCAGCAG TCAGCAATGC TTATCTCCTC GCCTTCCTTA TCATCAGTAG CTAGGACAGT AGCTTGTGTG GATGG-----
SP803280_149_N10     TACGCAGCAG TCAGCAATGC TTATCTCCTC GCCTTCCTTA TCATCAGTAG CTAGGACAGT AGCTTGTGTG GATGGTTGGC
SP803280_257_A23     TACGCAGCAG TCAGCAATGC TTATCTCCTC GCCTTCCTTA TCATCAGTAG CTAGGACAGT AGCTTGTGTG GATGG-----
SP803280_453_B01     TACGCAGCAG TCAGCAATGC TTATCTCCTC GCCTTCCTTA TCATCAGTAG CTAGGACAGT AGCTTGTGTG GATGG-----
SP803280_494_M23     TACGCAGCAG TCAGCAATGC TTATCTCCTC GCCTTCCTTA TCATCAGTAG CTAGGACAGT AGCTTGTGTG GATGG-----
SP803280_524_N01     TACGCAGCAG TCAGCAATGC TTATCTCCTC GCCTTCCTTA TCATCAGTAG CTAGGACAGT AGCTTGTGTG GATGG-----

                     401
R570_104_G22         ---TTGGCCC TGGCTGTCAA AACCAAATGC TGGTACAGAC GTACAGTTGC TTCTAACGAA GTGTATATAT TAAGGTGGGA
R570_237_G04         ---TTGGCCC TGGCTGTCAA AACCAAATGC TGGTACAGAC GTACAGTTGC TTCTAACGAA GTGTATATAT TAAGGTGGGA
R570_096_D24         ---TTGGCCC TGGCTGTCAA AACCAAATGC TGGTACAGAC GTACAGTTGC TTCTAACGAA GTGTATATAT TAAGGTGGGA
R570_085_J02         ---TTGGCCC TGGCTGTCAA AACCAAATGC TGGTACAGAC GTACAGTTGC TTCTAACGAA GTGTATATAT TAAGGTGGGA
SP803280_264_P08     ---TTGGCCC TGGCTGTCAA AACCAAATGC TGGTACAGAC GTACAGTTGC TTCTAACGAA GTGTATATAT TAAGGTGGGA
SP803280_480_A02     ---TTGGCCC TGGCTGTCAA AACCAAATGC TGGTACAGAC GTACAGTTGC TTCTAACGAA GTGTATATAT TAAGGTGGGA
SP803280_548_I17     ---TTGGCCC TGGCTGTCAA AACCAAATGC TGGTACAGAC GTACAGTTGC TTCTAACGAA GTGTATATAT TAAGGTGGGA
SP803280_109_H09     ---TTGGCCC TGGCTGTCAA AACCAAATGC TGGTACAGAC GTACAGTTGC TTCTAACGAA GTGTATATAT TAAGGTGGGA
SP803280_264_H19     ---TTGGCCC TGGCTGTCAA AACCAAATGC TGGTACAGAC GTACAGTTGC TTCTAACGAA GTGTATATAT TAAGGTGGGA
SP803280_228_N18     ---TTGGCCC TGCCTGTCAA AACCAAATGC TGGTACAGAC GTACAGTTGC TTCTAACGAA GTGTATATAT TAAGGTGGGA
SP803280_441_A04     ---TTGGCCC TGGCTGTCAA AACCAAATGC TGGTACAGAC GTACAGTTGC TTCTAACGAA GTGTATATAT TAAGGTGGGA
SP803280_258_B07     ---TTGGCCC TGGCTGTCAA AACCAAATGC TGGTACAGAC GTACAGTTGC TTCTAACGAA GTGTATATAT TAAGGTGGGA
SP803280_273_J18     ---TTGGCCC TGGCTGTCAA AACCAAATGC TGGTACAGAC GTACAGTTGC TTCTAACGAA GTGTATATAT TAAGGTGGGA
SP803280_465_H15     ---TTGGCCC TGGCTGTCAA AACCAAATGC TGGTACAGAC GTACAGTTGC TTCTAACGAA GTGTATATAT TAAGGTGGGA
SP803280_456_J23     ---TTGGCCC TGGCTGTCAA AACCAAATGC TGGTACAGAC GTACAGTTGC TTCTAACGAA GTGTATATAT TAAGGTGGGA
SP803280_492_H21     ---TTGGCCC TGGCTGTCAA AACCAAATGC TGGTACAGAC GTACAGTTGC TTCTAACGAA GTGTATATAT TAAGGTGGGA
SP803280_149_N10     CACTTGGCCC TGGCTGTCAA AACCAAATGC TGGTACAGAC GTACAGTTGC TTCTAACGAA GTGTATATAT TAAGGTGGGA
SP803280_257_A23     ---TTGGCCC TGGCTGTCAA AACCAAATGC TGGTACAGAC GTACAGTTGC TTCTAACGAA GTGTATATAT TAAGGTGGGA
SP803280_453_B01     ---TTGGCCC TGGCTGTCAA AACCAAATGC TGGTACAGAC GTACAGTTGC TTCTAACGAA GTGTATATAT TAAGGTGGGA
SP803280_494_M23     ---TTGGCCC TGGCTGTCAA AACCAAATGC TGGTACAGAC GTACAGTTGC TTCTAACGAA GTGTATATAT TAAGGTGGGA
SP803280_524_N01     ---TTGGCCC TGGCTGTCAA AACCAAATGC TGGTACAGAC GTACAGTTGC TTCTAACGAA GTGTATATAT TAAGGTGGGA

                     481
R570_104_G22         GGAGATACTA CCTGTCGATG CAGCCATCCT TTACTTCTTC TTTTTGCCCC CACAAAGCAA GACTTTATCA GTTGTTGACC
R570_237_G04         GGAGATACTA CCTGTCGATG CAGCCATCCT TTACTTCTTC TTTTTGCCCC CACAAAGCAA GACTTTATCA GTTGTTGACC
R570_096_D24         GGAGATACTA CCTGTCGATG CAGCCATCCT TTACTTCTTC TTTTTGCCCC CACAAAGCAA GACTTTATCA GTTGTTGACC
R570_085_J02         GGAGATACTA CCTGTCGATG CAGCCATCCT TTACTTCTTC TTTTTGCCCC CACAAAGCAA GACTTTATCA GTTGTTGACC
SP803280_264_P08     GGAGATACTA CCTGTCGATG CAGCCATCCT TTACTTCTTC TTTTTGCCCC CACAAAGCAA GACTTTATCA GTTGTTGACC
SP803280_480_A02     GGAGATACTA CCTGTCGATG CAGCCATCCT TTACTTCTTC TTTTTGCCCC CACAAAGCAA GACTTTATCA GTTGTTGACC
SP803280_548_I17     GGAGATACTA CCTGTCGATG CAGCCATCCT TTACTTCTTC TTTTTGCCCC CACAAAGCAA GACTTTATCA GTTGTTGACC
SP803280_109_H09     GGAGATACTA CCTGTCGATG CAGCCATCCT TTACTTCTTC TTTTTGCCCC CACAAAGCCA GACTTTATCA GTTGTTGACC
SP803280_264_H19     GGAGATACTA CCTGTCGATG CAGCCATCCT TTACTTCTTC TTTTTGCCCC CACAAAGCCA GACTTTATCA GTTGTTGACC
SP803280_228_N18     GGAGATACTA CCTGTCGATG CAGCCATCCT TTACTTCTTC TTTTTGCCCC CACAAAGCAA GACTTTATCA GTTGTTGACC
SP803280_441_A04     GGAGATACTA CCTGTCGATG CAGCCATCCT TTACTTCTTC TTTTTGCCCC CACAAAGCAA GACTTTATCA GTTGTTGACC
SP803280_258_B07     GGAGATACTA CCTGTCGATG CAGCCATCCT TTACTTCTTC TTTTTGCCCC CACAAAGCAA GACTTTATCA GTTGTTGACC
SP803280_273_J18     GGAGATACTA CCTGTCGATG CAGCCATCCT TTACTTCTTC TTTTTGCCCC CACAAAGCAA GACTTTATCA GTTGTTGACC
SP803280_465_H15     GGAGATACTA CCTGTCGATG CAGCCATCCT TTACTTCTTC TTTTTGCCCC CACAAAGCAA GACTTTATCA GTTGTTGACC
SP803280_456_J23     GGAGATACTA CCTGTCGATG CAGCCATCCT TTACTTCTTC TTTTTGCCCC CACAAAGCAA GACTTTATCA GTTGTTGACC
SP803280_492_H21     GGAGATACTA CCTGTCGATG CAGCCATCCT TTACTTCTTC TTTTTGCCCC CACAAAGCAA GACTTTATCA GTTGTTGACC
SP803280_149_N10     GGAGATACTA CCTGTCGATG CAGCCATCCT TTACTTCTTC TTTTTGCCCC CACAAAGCAA GACTTTATCA GTTGTTGACC
SP803280_257_A23     GGAGATACTA CCTGTCGATG CAGACATCCT TTACTTCTTC TTTTTGCCCC CACAAAGCAA GACTTTATCA GTTGTTGACC
SP803280_453_B01     GGAGATACTA CCTGTCGATG CAGACATCCT TTACTTCTTC TTTTTGCCCC CACAAAGCAA GACTTTATCA GTTGTTGACC
SP803280_494_M23     GGAGATACTA CCTGTCGATG CAGACATCCT TTACTTCTTC TTTTTGCCCC CACAAAGCAA GACTTTATCA GTTGTTGACC
SP803280_524_N01     GGAGATACTA CCTGTCGATG CAGACATCCT TTACTTCTTC TTTTTGCCCC CACAAAGCAA GACTTTATCA GTTGTTGACC

                     561
R570_104_G22         AAATGGCAGC TCAAAAATAA GAAGCACATT GTTGTGTTTG CCGTGTTTGT TCAGGGAAGT ---------- ----------
R570_237_G04         AAATGGCAGC TCAAAAATAA GAAGCACATT GTTGTGTTTG CCGTGTTTGT TCAGGGAAGT ---------- ----------
R570_096_D24         AAATGGCAGC TCAAAAATAA GAAGCACATT GTTGTGTTTG CCGTGTTTGT TCAGGGAAGT ---------- ----------
R570_085_J02         AAATGGCAGC TCAAAAATAA GAAGCACATT GTTGTGTTTG CCGTGTTTGT TCAGGGAAGT ---------- ----------
SP803280_264_P08     AAATGGCAGC TCAAAAATAA GAAGCACATT GTTGTGTTTG CCGTGTTTGT TCAGGGAAGT ---------- ----------
SP803280_480_A02     AAATGGCAGC TCAAAAATAA GAAGCACATT GTTGTGTTTG CCGTGTTTGT TCAGGGAAGT ---------- ----------
SP803280_548_I17     AAATGGCAGC TCAAAAATAA GAAGCACATT GTTGTGTTTG CCGTGTTTGT TCAGGGAAGT ---------- ----------
SP803280_109_H09     AAGTGGCAGC TCAAAAATAA GGAGCACATT GTTGTGTTTG CCGTGTTTGT CCAGGGAAGT ---------- ----------
SP803280_264_H19     AAGTGGCAGC TCAAAAATAA GGAGCACATT GTTGTGTTTG CCGTGTTTGT CCAGGGAAGT ---------- ----------
SP803280_228_N18     AAATGGCAGC TCAAAAATAA GAAGCACATT GTTGTGTTTG CCGTGTTTGT TCAGGGAAGT ---------- ----------
SP803280_441_A04     AAATGGCAGC TCAAAAATAA GAAGCACATT GTTGTGTTTG CCGTGTTTGT TCAGGGAAGT ---------- ----------
SP803280_258_B07     AAATGGCAGC TCAAAAATAA GAAGCACATT GTTGTGTTTG CCGTGTTTGT TCAGGGAAGT ---------- ----------
SP803280_273_J18     AAATGGCAGC TCAAAAATAA GAAGCACATT GTTGTGTTTG CCGTGTTTGT TCAGGGAAGT ---------- ----------
SP803280_465_H15     AAATGGCAGC TCAAAAATAA GAAGCACATT GTTGTGTTTG CCGTGTTTGT TCAGGGAAGT ---------- ----------
SP803280_456_J23     AAATGGCAGC TCAAAAATAA GAAGCACATT GTTGTGTTTG CCGTGTTTGT TCAGGGAAGT ---------- ----------
SP803280_492_H21     AAATGGCAGC TCAAAAATAA GAAGCACATT GTTGTGTTTG CCGTGTTTGT TCAGGGAAGT ---------- ----------
SP803280_149_N10     AAATGGCAGC TCAAAAATAA GAAGCACATT GTTGTGTTTG CCGTGTTTGT TCAGGGAAGT TTAGGCCGCG TTCACTTGGC
SP803280_257_A23     AAATGGCAGC TCAAAAATAA GAAGCACATT GTTGTGTTTG CCGTGTTTGT TCAGGGAAGT ---------- ----------
SP803280_453_B01     AAATGGCAGC TCAAAAATAA GAAGCACATT GTTGTGTTTG CCGTGTTTGT TCAGGGAAGT ---------- ----------
SP803280_494_M23     AAATGGCAGC TCAAAAATAA GAAGCACATT GTTGTGTTTG CCGTGTTTGT TCAGGGAAGT ---------- ----------
SP803280_524_N01     AAATGGCAGC TCAAAAATAA GAAGCACATT GTTGTGTTTG CCGTGTTTGT TCAGGGAAGT ---------- ----------
```

```
                 641
R570_104_G22       ---------- ---------- ---------- ---------- ---------- ---------- ---------- ----------
R570_237_G04       ---------- ---------- ---------- ---------- ---------- ---------- ---------- ----------
R570_096_D24       ---------- ---------- ---------- ---------- ---------- ---------- ---------- ----------
R570_085_J02       ---------- ---------- ---------- ---------- ---------- ---------- ---------- ----------
SP803280_264_P08   ---------- ---------- ---------- ---------- ---------- ---------- ---------- ----------
SP803280_480_A02   ---------- ---------- ---------- ---------- ---------- ---------- ---------- ----------
SP803280_548_I17   ---------- ---------- ---------- ---------- ---------- ---------- ---------- ----------
SP803280_109_H09   ---------- ---------- ---------- ---------- ---------- ---------- ---------- ----------
SP803280_264_H19   ---------- ---------- ---------- ---------- ---------- ---------- ---------- ----------
SP803280_228_N18   ---------- ---------- ---------- ---------- ---------- ---------- ---------- ----------
SP803280_441_A04   ---------- ---------- ---------- ---------- ---------- ---------- ---------- ----------
SP803280_258_B07   ---------- ---------- ---------- ---------- ---------- ---------- ---------- ----------
SP803280_273_J18   ---------- ---------- ---------- ---------- ---------- ---------- ---------- ----------
SP803280_465_H15   ---------- ---------- ---------- ---------- ---------- ---------- ---------- ----------
SP803280_456_J23   ---------- ---------- ---------- ---------- ---------- ---------- ---------- ----------
SP803280_492_H21   ---------- ---------- ---------- ---------- ---------- ---------- ---------- ----------
SP803280_149_N10   ATGCATACCC AAAATATTTC CTCTTCCACC CATCACATCG AATCTTTAGA TGCATGCATG AAGCATTAAA TGTAGGTAAA
SP803280_257_A23   ---------- ---------- ---------- ---------- ---------- ---------- ---------- ----------
SP803280_453_B01   ---------- ---------- ---------- ---------- ---------- ---------- ---------- ----------
SP803280_494_M23   ---------- ---------- ---------- ---------- ---------- ---------- ---------- ----------
SP803280_524_N01   ---------- ---------- ---------- ---------- ---------- ---------- ---------- ----------

                 721
R570_104_G22       ---------- ---------- ---------- ---------- ---------- ---------- ---------- ----------
R570_237_G04       ---------- ---------- ---------- ---------- ---------- ---------- ---------- ----------
R570_096_D24       ---------- ---------- ---------- ---------- ---------- ---------- ---------- ----------
R570_085_J02       ---------- ---------- ---------- ---------- ---------- ---------- ---------- ----------
SP803280_264_P08   ---------- ---------- ---------- ---------- ---------- ---------- ---------- ----------
SP803280_480_A02   ---------- ---------- ---------- ---------- ---------- ---------- ---------- ----------
SP803280_548_I17   ---------- ---------- ---------- ---------- ---------- ---------- ---------- ----------
SP803280_109_H09   ---------- ---------- ---------- ---------- ---------- ---------- ---------- ----------
SP803280_264_H19   ---------- ---------- ---------- ---------- ---------- ---------- ---------- ----------
SP803280_228_N18   ---------- ---------- ---------- ---------- ---------- ---------- ---------- ----------
SP803280_441_A04   ---------- ---------- ---------- ---------- ---------- ---------- ---------- ----------
SP803280_258_B07   ---------- ---------- ---------- ---------- ---------- ---------- ---------- ----------
SP803280_273_J18   ---------- ---------- ---------- ---------- ---------- ---------- ---------- ----------
SP803280_465_H15   ---------- ---------- ---------- ---------- ---------- ---------- ---------- ----------
SP803280_456_J23   ---------- ---------- ---------- ---------- ---------- ---------- ---------- ----------
SP803280_492_H21   ---------- ---------- ---------- ---------- ---------- ---------- ---------- ----------
SP803280_149_N10   AAAATAACTA ATTGCATAGT TTGTCTGTAA AGTTCGAAAC GAATCTTTTG AGTCTAATTA ATCCATGGTT GGACAAATTT
SP803280_257_A23   ---------- ---------- ---------- ---------- ---------- ---------- ---------- ----------
SP803280_453_B01   ---------- ---------- ---------- ---------- ---------- ---------- ---------- ----------
SP803280_494_M23   ---------- ---------- ---------- ---------- ---------- ---------- ---------- ----------
SP803280_524_N01   ---------- ---------- ---------- ---------- ---------- ---------- ---------- ----------

                 801
R570_104_G22       ---------- ---------- ---------- ---------- ---------- ---------- ----TTACAG TCAGAATCAC
R570_237_G04       ---------- ---------- ---------- ---------- ---------- ---------- ----TTACAG TCAGAATCAC
R570_096_D24       ---------- ---------- ---------- ---------- ---------- ---------- ----TTACAG TCAAAATCAC
R570_085_J02       ---------- ---------- ---------- ---------- ---------- ---------- ----TTACAG TCAGAATCAC
SP803280_264_P08   ---------- ---------- ---------- ---------- ---------- ---------- ----TTACAG TCAAAATCAC
SP803280_480_A02   ---------- ---------- ---------- ---------- ---------- ---------- ----TTACAG TCAAAATCAC
SP803280_548_I17   ---------- ---------- ---------- ---------- ---------- ---------- ----TTACAG TCAAAATCAC
SP803280_109_H09   ---------- ---------- ---------- ---------- ---------- ---------- ----GTACAG TCAAAATCAC
SP803280_264_H19   ---------- ---------- ---------- ---------- ---------- ---------- ----GTACAG TCAAAATCAC
SP803280_228_N18   ---------- ---------- ---------- ---------- ---------- ---------- ----TTACAG TCAAAATCAC
SP803280_441_A04   ---------- ---------- ---------- ---------- ---------- ---------- ----TTACAG TCAGAATCAC
SP803280_258_B07   ---------- ---------- ---------- ---------- ---------- ---------- ----TTACAG TCAGAATCAC
SP803280_273_J18   ---------- ---------- ---------- ---------- ---------- ---------- ----TTACAG TCAGAATCAC
SP803280_465_H15   ---------- ---------- ---------- ---------- ---------- ---------- ----TTACAG TCAGAATCAC
SP803280_456_J23   ---------- ---------- ---------- ---------- ---------- ---------- ----TTACAG TCAGAATCAC
SP803280_492_H21   ---------- ---------- ---------- ---------- ---------- ---------- ----TTACAG TCAGAATCAC
SP803280_149_N10   TACCAAATAA CAACGAAACT GCTACAGTAA CTTTTCACCC AAATTTTCTC AACTAAACAC AGCCTTACAG TCAAAATCAC
SP803280_257_A23   ---------- ---------- ---------- ---------- ---------- ---------- ----TTACAG ACAAAATCAC
SP803280_453_B01   ---------- ---------- ---------- ---------- ---------- ---------- ----TTACAG ACAAAATCAC
SP803280_494_M23   ---------- ---------- ---------- ---------- ---------- ---------- ----TTACAG ACAAAATCAC
SP803280_524_N01   ---------- ---------- ---------- ---------- ---------- ---------- ----TTACAG ACAAAATCAC

                 881
R570_104_G22       TTCTTCTTCT TCTTCCTCCT CTTTTACAGC TGAGTTGTGC CAGTCTGCCA GATG
R570_237_G04       TTCTTCTTCT TCTTCCTCCT CTTTTACAGC TGAGTTGTGC CAGTCTGCCA GATG
R570_096_D24       TTCTTCTTCT TCTTCCTCCT CTTTTACAGC TGAGTTGTGC CAGTCTGCCA GATG
R570_085_J02       TTCTTCTTCT TCTTCCTCCT CTTTTACAGC TGAGTTGTGC CAGTCTGCCA GATG
SP803280_264_P08   TTCTTCTTCT TCTTCCTCCT CTTTTACAGC TGAGTTGTGC CAGTCTGCCA GATG
SP803280_480_A02   TTCTTCTTCT TCTTCCTCCT CTTTTACAGC TGAGTTGTGC CAGTCTGCCA GATG
SP803280_548_I17   TTCTTCTTCT TCTTCCTCCT CTTTTACAGC TGAGTTGTGC CAGTCTGCCA GATG
SP803280_109_H09   TTCTTCTTCT CCTTCTTCCT CTTTTACAGC TGAGTTGTGC CAGTGTGCCA GATG
SP803280_264_H19   TTCTTCTTCT CCTTCTTCCT CTTTTACAGC TGAGTTGTGC CAGTGTGCCA GATG
SP803280_228_N18   TTCTTCTTCT TCTTCCTCCT CTTTTACAGC TGAGTTGTGC CAGTCTGCCA GATG
SP803280_441_A04   TTCTTCTTCT TCTTCCTCCT CTTTTACAGC TGAGTTGTGC CAGTCTGCCA GATG
SP803280_258_B07   TTCTTCTTCT TCTTCCTCCT CTTTTACAGC TGAGTTGTGC CAGTCTGCCA GATG
SP803280_273_J18   TTCTTCTTCT TCTTCCTCCT CTTTTACAGC TGAGTTGTGC CAGTCTGCCA GATG
SP803280_465_H15   TTCTTCTTCT TCTTCCTCCT CTTTTACAGC TGAGTTGTGC CAGTCTGCCA GATG
SP803280_456_J23   TTCTTCTTCT TCTTCCTCCT CTTTTACAGC TGAGTTGTGC CAGTCTGCCA GATG
SP803280_492_H21   TTCTTCTTCT TCTTCCTCCT CTTTTACAGC TGAGTTGTGC CAGTCTGCCA GATG
SP803280_149_N10   TTCTTCTTCT TCTTCCTCCT CTTTTACAGC TGAGTTGTGC CAGTCTGCCA GATG
SP803280_257_A23   TTCTTCTT-- -CTTCCTCCT CTTTTACAGC TGAGTTGTGC CAGTCTGCCA GATG
SP803280_453_B01   TTCTTCTT-- -CTTCCTCCT CTTTTACAGC TGAGTTGTGC CAGTCTGCCA GATG
SP803280_494_M23   TTCTTCTT-- -CTTCCTCCT CTTTTACAGC TGAGTTGTGC CAGTCTGCCA GATG
SP803280_524_N01   TTCTTCTT-- -CTTCCTCCT CTTTTACAGC TGAGTTGTGC CAGTCTGCCA GATG
```
