# Supplementary material for: Sucrose-phosphate phosphatase from sugarcane reveals an ancestral tandem duplication
Source: BMC Plant Biol. 2021 Jan 7;21:23. doi: 10.1186/s12870-020-02795-5 (PMC7792115; doi:10.1186/s12870-020-02795-5)
Supplement: Supplementary file 4 — Additional file 4: Supplementary Figure 4. Poaceae S6PP domain multiple alignement. [file 12870_2020_2795_MOESM4_ESM.pdf]

```
Alignment: C:\Users\olgui\OneDrive\Documentos\S6PP_Phytozome\Alinhamento_DominioS6PP_Poaceaes_atualizado-07-2020_Refinado.fas
Seaview [blocks=10 fontsize=6 A4] on Thu Aug 13 15:57:04 2020

                                                        1
B.distachyon-v3.2_Bradi1g55300.2.p                      ARLMIVSDLD HTMVDHHDEE NLSLLR--FG ALWESLYCQD SLLVFSTGRS PTLYKELRKE KPMLTPDITI MSVGTEITYG
B.distachyon-v3.2_Bradi2g36350.6.p                      ARLMIVSDLD QTMVDHCDPE NSALLR--FE ALWESEYSQD SLLVFSTGRS PVSYRGIRKE KPLLTPDITI MSVGTVIAYG
B.hybridum-v1.1_Brahy.D01G0746500.1.p                   ARLMIVSDLD HTMVDHHDEE NLSLLR--FG ALWESLYCQD SLLVFSTGRS PTLYKELRKE KPMLTPDITI MSVGTEITYG
B.hybridum-v1.1_Brahy.D02G0478800.1.p                   ARLMIVSDLD QTMVDHCDPE NSALLR--FE ALWESEYSQD SLLVFSTGRS PVSYRGIRKE KPLLTPDITI MSVGTVIAYG
B.hybridum-v1.1_Brahy.S06G0207800.1.p                   ARLMIVSDLD HTMVDHHDEE NLSLLR--FG ALWESVYCQD SLLVFSTGRS PTLYKELRKE KPMLTPDITI MSVGTEITYG
B.hybridum-v1.1_Brahy.S08G0229700.1.p                   ARLMIVSDLD QTMVDHCDPE NSALLR--FD VLWESEYSQD SLLVFSTGRS PVSYRGIRKE KPLLTPDIAI MSVGTVIAYG
B.stacei-v1.1_Brast06G189100.1.p                        ARLMIVSDLD HTMVDHHDEE NLSLLR--FG ALWESVYCQD SLLVFSTGRS PTLYKELRKE KPMLTPDITI MSVGTEITYG
B.stacei-v1.1_Brast08G210900.1.p                        ARLMIVSDLD QTMVDHRDPE NSALLR--FD VLWESEYSQD SLLVFSTGRS PVSYRGIRKE KPLLTPDIAI MSVGTVIAYG
B.sylvaticum-v1.1_Brasy6G197100.1.p                     ARLMIVSDLD HTMVDHHDEE NLSLLR--FG ALWESLYCQD SLLVFSTGRS PTLYKELRKE KPMLTPDITI MSVGTEITYG
B.sylvaticum-v1.1_Brasy8G226900.1.p                     ARLMIVSDLD QTLVDHCDPE NSALLR--FE ALWESEYSQD SLLVFSTGRS PVSYRGLRKE KPLLTPDITI MSVGTVISYG
E.coracana-v1.1_ELECO.r07.1AG0017250.1                  ARLMIVSDLD HTMVDHHDEE NLSLLR--FG SLWESVYCQD SLLVFSTGRS PTLYKELRKE KPMLTPDITI MSVGTEITYG
E.coracana-v1.1_ELECO.r07.1BG0066280.1                  ARLMIVSDLD HTMVDHHDEE NLSLLR--FG ALWESVYCQD SLLVFSTGRS PTLYKELRKE KPMLTPDITI MSVGTEITYG
E.coracana-v1.1_ELECO.r07.5AG0405240.1                  ARLMIVSDLD QTMVDHEDPD NLSLLS--FQ ALWKAEYSQD SLLVFSTGRT PVSYKRLRKE KPLITPDITI MSVGTVIAYG
E.coracana-v1.1_ELECO.r07.5BG0453340.1                  ARLMIVSDLD QTMVDHEDPD NLSLLS--FQ ALWKAEYSQD SLLVFSTGRS PVSYKRLGKD KPLITPDITI MSVGTVIAYG
H.vulgare-r1_HORVU1Hr1G019500.3                         ARLMIVSDLD QTMVDHSDPE SSAMLR--FE ALWESEYSQD SLLVFSTGRT PVSYKGLRKI KPLLTPDITI MSVGTVIAYG
H.vulgare-r1_HORVU5Hr1G000580.1                         ARLMIVSDLD HTMVDHHDEE NLSLLR--FG ALWESVYCQD SLLVFSTGRS PTLYKELRKE KPMLTPDITI MSVGSEITYG
M.sinensis-v7.1_Misin07G375700.1.p                      ARLMIVSDLD HTMVDHHDEE NLSLLR--FG ALWESVYCED SLLVFSTGRS PTLYKELRKE KPMLTPDITI MSVGTEITYG
M.sinensis-v7.1_Misin16G055500.1.p_S6PP.1               ARLVLVSDLD QTMIDHQDRE NLSLLR--FE ALWEAEFSQD SLLVFSTGRT PISYKGLRKD KPLITPDITI MSVGTVIAYG
M.sinensis-v7.1_Misin16G055600.1.p_S6PP.2               ARLMIVSNLD QTMVDHDDPE DLSLLR--FE ALWEAEFAHD SLLIFSTGRS PISYSDLRKN KPLITPDITI MSVGTVIAYG
M.sinensis-v7.1_Misin17G051300.1.p_S6PP.2               ARLMIVSDLD QTMVDHDDPE DLSLLR--FE ALWEAEFSHD SLLIFSTGRS PISYHDLRKN KPLITPDITI MSVGTVIAYG
M.sinensis-v7.1_Misin17G051400.1.p_S6PP.1               ARLVLVSDLD QTMIDHQDQE NLSLLR--FE ALWEAEFSQD SLLVFSTGRT PISYKGLRKD KPLITPDITI MSVGTVIAYG
O.sativaKitaake-v3.1_OsKitaake01g172900.1.p             ARLIIVSDLD HTMVDHHDEE NLSLLR--FG ALWESVYCQD SLLVFSTGRS PTLYKELRKE KPMLTPDITI MSVGTEITYG
O.sativaKitaake-v3.1_OsKitaake02g035100.1.p             ARLMIVSDLD HTMVDHHDEE NLSLLR--FG ALWESVYCQD SLLVFSTGRS PTLYMELRKE KPMLTPDITI MSVGTEITYG
O.sativaKitaake-v3.1_OsKitaake05g032900.1.p             ARLMIVSDLD QTMIDHNDPK NLSLLR--FQ ALWESEFSQD SLLVFSTGRS PISYRGLRTQ KPLITPDITI MSVGTVIAYG
O.sativa-v7.0_LOC_Os01g27880.1                          ARLIIVSDLD HTMVDHHDEE NLSLLR--FG ALWESVYCQD SLLVFSTGRS PTLYKELRKE KPMLTPDITI MSVGTEITYG
O.sativa-v7.0_LOC_Os02g05030.1                          ARLMIVSDLD HTMVDHHDEE NLSLLR--FG ALWESVYCQD SLLVFSTGRS PTLYMELRKE KPMLTPDITI MSVGTEITYG
O.sativa-v7.0_LOC_Os05g05270.1                          ARLMIVSDLD QTMIDHNDPK NLSLLR--FQ ALWESEFSQD SLLVFSTGRS PISYRGLRTQ KPLITPDITI MSVGTVIAYG
O.thomaeum-v1.0_Oropetium_20150105_15513A               ARLMIVSDLD QTMVDHDDPE NQSLLS--FE ALWEAEYSQD CLLIFSTGRS PVSYKRLRKE KPLITPDITI MSVGTIIAY-
O.thomaeum-v1.0_Oropetium_20150105_22356A               ARLMIVSDLD HTMVDHHDEE NLSLLR--FG ALWESVYCQD SLLVFSTGRS PTLYKELRKE KPMLTPDITI MSVGTEITYG
P.halliiHAL-v2.1_PhHAL.3G110000.1.p_S6PP.1              ARLVIVSDLD QTMIDHDDRE NLSLLR--FE ALWEAEFSRD SLLVFSTGRT PISYKGLRKE KPLITPDITI MSVGTVIAYG
P.halliiHAL-v2.1_PhHAL.3G110100.1.p_S6PP.2              ARLMIVSDLD QTMVDHDDPE DLSLLR--FG ALWEAEFAHD SLLIFSTGRS LISYKDLRKE KPLVTPDITV MSVGTVIAYG
P.halliiHAL-v2.1_PhHAL.5G367300.1.p                     ARLMIVSDLD HTMVDHHDEE NLSLLR--FA ALWESTYCQD SLLVFSTGRS PTLYKELRKE KPMLTPDITI MSVGTEITYG
P.hallii-v3.1_Pahal.3G115900.1.p_S6PP.1                 ARLVIVSDLD QTMIDHDDRE NLSLLR--FE ALWEAEFSRN SLLVFSTGRT PISYKGLRKE KPLITPDITI MSVGTVIAYG
P.hallii-v3.1_Pahal.3G116000.1.p_S6PP.2                 ARLMIVSDLD QTMVDHDDPE DLSLLR--FG ALWEAEFAHD SLLIFSTGRS LISYKDLRKE KPLVTPDITV MSVGTVIAYG
P.hallii-v3.1_Pahal.5G373100.1.p                        ARLMIVSDLD HTMVDHHDEE NLSLLR--FA ALWESTYCQD SLLVFSTGRS PTLYKELRKE KPMLTPDITI MSVGTEITYG
P.virgatum-v5.1_Pavir.3KG143200.2.p                     ASLVIVSDLD QTMIDHDDRE NLSLLR--FE ALWEAEFSQD SLLVFSTGRT PISYKGLQKD KPLITPDITI MSVGTVIAYG
P.virgatum-v5.1_Pavir.3NG188964.1.p_S6PP.1              ARLVIVSDLD QTMIDHDDRE NLSLLR--FE ALWEAEFSQD SLLVFSTGRT PISYKGLRKD KPLITPDITI MSVGTVIAYG
P.virgatum-v5.1_Pavir.3NG189313.1.p_S6PP.2              ARLMIVSDLD QTMVDHDDPE DLSLLR--FG ALWEAEFAHD YLLIFSTGRS LISYKDLRKE KPLITPDITV MSVGTVIAYG
P.virgatum-v5.1_Pavir.5KG244800.2.p                     ARLMIVSDLD HTMVDHHDEE NLSLLR--FA ALWESIYCQD SLLVFSTGRS PTLYKELRKE KPMLTPDITI MSVGTEITYG
P.virgatum-v5.1_Pavir.5NG369700.2.p                     ARLMIVSDLD HTMVDHHDEE NLSLLR--FA ALWESVYCQD SLLVFSTGRS PTLYKELRKE KPMLTPDITI MSVGTEITYG
S.bicolor_RTx430-v2.1_SbiRTX430.04G160000.1.p           ARLMIVSDLD HTMVDHHDEE NLSLLR--FG ALWESVYCQD SLLVFSTGRS PTLYKELRKE KPMLTPDITI MSVGTEITYG
S.bicolor_RTx430-v2.1_SbiRTX430.09G042400.1.p_S6PP.1    ARLMLVSDLD QTMIDHQDRE NLSLLR--FE ALWEAEFSQD SLLVFSTGRT PISYKGLRKD KPLITPDITI MSVGTVIAYG
S.bicolor_RTx430-v2.1_SbiRTX430.09G042500.1.p_S6PP.2    ARLMIVSDLD QTMVDHDDPE DLSLLR--FE ALWEAEFSHD SLLIFSTGRS PISYNDLRKN KPLITPDITI MSVRTVIAYG
S.bicolorRio-v2.1_SbRio.04G161300.1.p                   ARLMIVSDLD HTMVDHHDEE NLSLLR--FG ALWESVYCQD SLLVFSTGRS PTLYKELRKE KPMLTPDITI MSVGTEITYG
S.bicolorRio-v2.1_SbRio.09G043600.1.p_S6PP.1            ARLMLVSDLD QTMIDHQDRE NLSLLR--FE ALWEAEFSQD SLLVFSTGRT PISYKGLRKD KPLITPDITI MSVGTVIAYG
S.bicolorRio-v2.1_SbRio.09G043700.1.p_S6PP.2            ARLMIVSDLD QTMVDHDDPE DLSLLS--FE ALWEAEFSHD SLLIFSTGRS PISYNDLRKN KPLITPDITI MSVGTVIAYG
S.bicolor-v3.1.1_Sobic.004G151800.2.p                   ARLMIVSDLD HTMVDHHDEE NLSLLR--FG ALWESVYCQD SLLVFSTGRS PTLYKELRKE KPMLTPDITI MSVGTEITYG
S.bicolor-v3.1.1_Sobic.009G040900.2.p_S6PP.1            ARLMLVSDLD QTMIDHQDRE NLSLLR--FE ALWEAEFSQD SLLVFSTGRT PISYKGLRKD KPLITPDITI MSVGTVIAYG
S.bicolor-v3.1.1_Sobic.009G041000.1.p_S6PP.2            ARLMIVSDLD QTMVDHDDPE DLSLLS--FE ALWEAEFSHD SLLIFSTGRS PISYNDLRKN KPLITPDITI MSVGTVIAYG
S.italica-v2.2_Seita.2G386000.1.p                       ARLMIVSDLD HTMVDHHDEE NLSLLR--FG ALWESIYCQD SLLVFSTGRS PTLYKELRKE KPMLTPDITI MSVGTEITYG
S.italica-v2.2_Seita.3G059500.1.p_S6PP.1                ARLVIVSDLD QTMIDHDDRE NLSLLR--FE ALWEAEFSQD SLLVFSTGRT PISYKGLRKE KPLITPDITI MSVGTVIAYG
S.italica-v2.2_Seita.3G059600.1.p_S6PP.2                ARLMIVSDLD QTMVDHDDPE DLSLLR--FG ALWEAEFSHD SLLVFSTGRS LISYKDLRKE KPLITPDITV MSVGTVIAYG
S.italica-v2.2_Seita.5G174500.1.p                       ARLMIVSDLD HTMVDHHDEE NLSLLR--FG ALWESVYCQD SLLVFSTGRS PTLYKELRKE KPMLTPDITI MSVGTEITYG
S.viridis-v2.1_Sevir.2G396500.2.p                       ARLMIVSDLD HTMVDHHDEE NLSLLR--FG ALWESIYCQD SLLVFSTGRS PTLYKELRKE KPMLTPDITI MSVGTEITYG
S.viridis-v2.1_Sevir.3G060400.2.p_S6PP.1                ARLVIVSDLD QTMIDHDDRE NLSLLR--FE ALWEAEFSQD SLLVFSTGRT PISYKGLRKE KPLITPDITI MSVGTVIAYG
S.viridis-v2.1_Sevir.5G174500.1.p                       ARLMIVSDLD HTMVDHHDEE NLSLLR--FG ALWESVYCQD SLLVFSTGRS PTLYKELRKE KPMLTPDITI MSVGTEITYG
Saccharum-R570_S6PP.1                                   ARLVLVSDLD QTMIDHQDRE NLSLLR--FE ALWEAEFSQD SLLVFSTGRT PISYKGLRKD KPLITPDITI MSVGTVIAYG
Saccharum-R570_S6PP.2                                   ARLMIVSDLD QTMVDHDDPE DLSLLR--FE ALWEAEFSHD SLLIFSTGRS PISYNDLRKN KPLITPDITI MSVGTVIAYG
Saccharum-R570_S6PP.2D.2                                ARLMIVSDLD QTMVDHDDPE DLSLLR--FE ALWEAEFSHD SLLIFSTGRS PISYNDLRKN KPLITPDITI MSVGTVIAYG
Saccharum-R570_S6PP-2D.1                                ARLVLVSDLD QTMIDHQDRE NLSLLR--FE ALWEAEFSQD SLLVFSTGRT PISYKGLRKD KPLITPDITI MSVGTVIAYG
T.aestivum-v2.2_Traes_1BS_998F2E806.2                   ARLMIVSDLD QTMVDHCDPE NSALLR--FE ALWESEYSQD SLLVFSTGRT PVSYKALRER KPLLTPDITI MSVGTVIAYG
T.aestivum-v2.2_Traes_1DS_9AE5A76AC.2                   HPRPTATLAS RHLVDHCDPE NSALLR--FE ALWESEYSQD SLLVFSTGRT PVSYKGLRKR KPLLTPDITI MSVGTVIAYG
T.aestivum-v2.2_Traes_5AS_962BCA20C.1                   ARLMIVSDLD HTMVDHHDEE NLSLLR--FG ALWESVYCQD SLLVFSTGRS PTLYKELRKE KPMLTPDITI LSVGSEITYG
T.aestivum-v2.2_Traes_5BS_F7853DBB6.2                   ARLMIVSDLD HTMVDHHDEE NLSLLR--FG ALWESVYCQD SLLVFSTGRS PTLYKELRKE KPMLTPDITI LSVGSEITYG
T.aestivum-v2.2_Traes_5DS_0B17FFD55.1                   ARLMIVSDLD HTMVDHHDEE NLSLLR--FG ALWESVYCQD SLLVFSTGRS PTLYKELRKE KPMLTPDITI LSVGSEITYG
T.intermedium-v2.1_Thint.01G0102700.1.p                 ARLMIVSDLD QTMVDHCDSE NSALLR--FE ALWESEYSQD SLLVFSTGRT PVSYKGLRKR KPLLTPDITI MSVGTVIAYG
T.intermedium-v2.1_Thint.02G0243300.1.p                 ARLMIVSDLD QTMVDHCDPE NSALLR--FE ALWESEYSQD SLLVFSTGRT PVSYKGLRKI KPLLTPDITI MSVGTVIAYG
T.intermedium-v2.1_Thint.03G0206200.1.p                 ARLMIVSDLD QTMVDHYDPE NSALLH--FE ALWESEYSQD SLLVFSTGRT PVSYKGLRKA KPLLTPDITI MSVGTVIAYG
T.intermedium-v2.1_Thint.05G0032000.1.p                 ARLMIVSDLD HTMVDHHDEE NLSLLR--FG ALWESVYCQD SLLVFSTGRS PTLYKELRKE KPMLTPDITI MSVGTEITYG
T.intermedium-v2.1_Thint.13G0028000.1.p                 ARLMIVSDLD HTMVDHHDEE NLSLLR--FG ALWESAYCQD SLLVFSTGRS PTLYKELRKE KPMLTPDITI MSVGSEITYG
T.intermedium-v2.1_Thint.14G0034900.1.p                 ARLMIVSDLD HTMVDHHDEE NLSLLR--FG ALWESVYCQD SLLVFSTGRS PTLYKELRKE KPMLTPDITI LSVGSEITYG
T.intermedium-v2.1_Thint.15G0012900.1.p                 ARLMIVSDLD HTMVDHHDEE NLSLLR--FG ALWESVYCQD SLLVFSTGRS PTLYKELRKE KPMLTPDITI LSVGSEITYG
T.intermedium-v2.1_Thint.V1674200.1.p                   ARLMIVSDLD HTMVDHHDEE NLSLLR--FG ALWESVYCQD SLLVFSAGRS PTLYKELRKE KPMLTPDITI MSVGTEITYG
Z.maysPHJ40-v1.1_ZmPHJ40.08G139200.1.p                  ARLMIVSDLD HTMVNVVQLI SWSALSYLFG ALWESVYCED SLLVFSTGRS PTLYKELRKE KPMLTPDITI MSVGTEITYG
Z.maysPHJ40-v1.1_ZmPHJ40.10G092800.1.p                  ARLLLVSDLD QTMIDHDDRE NLSLLR--FE ALWEAEFAQD SLLVFSTGRT PVSYKGLRRD KPLVTPDVTI MSVGTVIAYG
```

```
                                                       81
B.distachyon-v3.2_Bradi1g55300.2.p                     EEMVPDAGWE EYLNNKWDKN IVLEETAKFP QL-KLQVESE QRPHKVSFLV DKKTAQEVIK SLSENFEKRG VDAKIIYSGG
B.distachyon-v3.2_Bradi2g36350.6.p                     EEMTPDSEWE EFLDNKWDRN VVAMETAKYP QL-KPQPERN QGPHKVSFFV DTQGAQEVIN SLPQKLEQFG LDVKIIYSSG
B.hybridum-v1.1_Brahy.D01G0746500.1.p                  EEMVPDAGWE EYLNNKWDKN IVLEETAKFP QL-KLQVESE QRPHKVSFLV DKKTAQEVIK SLSENFEKRG VDAKIIYSGG
B.hybridum-v1.1_Brahy.D02G0478800.1.p                  EEMTPDSEWE EFLDNKWDRN VVAMETAKYP QL-KPQPERN QGPHKVSFFV DTQGAQEVIN SLPQKLEQFG LDVKIIYSSG
B.hybridum-v1.1_Brahy.S06G0207800.1.p                  EEMVPDAGWE EYLNNKWDKN IVLEETAKFP QL-KLQVETE QRPHKVSFLV DKKTAQEVIK SLSENFEKRG VDAKIIYSGG
B.hybridum-v1.1_Brahy.S08G0229700.1.p                  EEMTPDAEWE VFLNNKWDRN VVAMETAKYP QL-KPQPERN QGPHKVSFFV DKQGAQQVIN SLPQKLEQCG LDVKIIYSSG
B.stacei-v1.1_Brast06G189100.1.p                       EEMVPDAGWE EYLNNKWDKN IVLEETAKFP QL-KLQVETE QRPHKVSFLV DKKTAQEVIK SLSENFEKRG VDAKIIYSGG
B.stacei-v1.1_Brast08G210900.1.p                       EEMTPDAEWE VFLNNKWDRN VVAMETAKYP QL-KPQPERN QGPHKVSFFV DKQGAQQVIN SLPQKLEQCG LDVKIIYSSG
B.sylvaticum-v1.1_Brasy6G197100.1.p                    EEMVPDAGWE EYLNNKWDKN IVLEETAKFP QL-KLQVESE QRPHKVSFLV DKKTAQEVIK SLSENFEKRG VDAKIIYSGG
B.sylvaticum-v1.1_Brasy8G226900.1.p                    EEMAPDAEWE EFLNNKWDRN VVAMETSKYP QL-KPQPERN QGPHKVSFFV DKQGAQEVIN SLPRKLEQCG LDVKIIYSSG
E.coracana-v1.1_ELECO.r07.1AG0017250.1                 EDMVPDEGWE EYLNNKWDRK IVVEETASFA EL-KLQPETE QRPHKVSFLV DKSNAQEVIK SLSEKLEKRG VDAKIIYSGG
E.coracana-v1.1_ELECO.r07.1BG0066280.1                 EDMVPDEGWE EYLNNKWDRK IVVEETASFA EL-KLQPETE QRPHKVSFLV DKSNAREVIK SLSEKLEKRG VDAKIIYSGG
E.coracana-v1.1_ELECO.r07.5AG0405240.1                 EEMICDIDWE EYLNNNWDRN IVVEETTKFP QL-KPQPKRS QGPHKVSFFV DKQGAQEVID SLPQKLQKRG LDVKIIYSSG
E.coracana-v1.1_ELECO.r07.5BG0453340.1                 EEMICDVDWE EYLNNNWDRN IVVEETTKFP QL-KPQPERS QGPHKVSFFV DKQGAQEVID SLPQKLQKRG LDVKIIYSSG
H.vulgare-r1_HORVU1Hr1G019500.3                        QEMTPDVGWE EFLNNKWDRD IVVQETATFP QL-KPQPERN QGPHKVSFFV DKQDAQEVIN SLPQKLEQLG LDVKIIYSSG
H.vulgare-r1_HORVU5Hr1G000580.1                        EAMVPDDGWE EYLNNKWDKN IVLEETAKFS EL-KLQPDTE QRPHKVSFLV DKKNAQGVIK SLSDKFEKRG VDAKIIYSGG
M.sinensis-v7.1_Misin07G375700.1.p                     EAMVPDDGWE EYLNNKWDRN IVVEETTSFS EL-KLQPETE QRPHKVSFFV DKKNAQEVIK SVAERLDKRG LDAKIIYSGG
M.sinensis-v7.1_Misin16G055500.1.p_S6PP.1              EEMIRDVGWE EYLNNNWDRT IVVEETARFS QL-KPQPERN QGPHKVSFFV DKQGAQEVMD YLPQKLEKRG LDVKIVYSNG
M.sinensis-v7.1_Misin16G055600.1.p_S6PP.2              VDMVRDVDWE EHLSSNWDRD IVVEEATKFP QL-KREPEKD QGPHKVTFVV EKQGAQEVMH ALPHKLKKHG IHVKIIFSYG
M.sinensis-v7.1_Misin17G051300.1.p_S6PP.2              GDMVRDADWE EHLSSNWDRD IVVEEAAKFP QL-KPEPEKD QGPHKVTFLV DKQGAQEVMH ALPHNLKKRG IHVKTIFSYG
M.sinensis-v7.1_Misin17G051400.1.p_S6PP.1              EEMIRDVGWE EYLNNNWDRT IVVEETARFP QL-KPQPERN QGPHKVSFFV DKQGAQEVMD YLPQKLEKRG LDVKIVYSSG
O.sativaKitaake-v3.1_OsKitaake01g172900.1.p            EAMVPDDGWE EYLNNKWDRN VVVEETAKFS EL-KLQPETE QRPHKVSFFV DKKSAQEVIK SLSGNMEKCG LDVKIIYSGG
O.sativaKitaake-v3.1_OsKitaake02g035100.1.p            EEMVPDDGWV EYLNNKWDRN IVVEETANVS EL-KLQVESE QRPHKVSFYV DKKSAQEVIK SLSEKLEKRG LDVKIIYSGG
O.sativaKitaake-v3.1_OsKitaake05g032900.1.p            EEMIHDVGWA EFLSNKWDRD IVVEETAKFP KL-KPQPERS QGPHKVSFFV DKEGAREVMD SLPETLNRRG LDVKIIFSSG
O.sativa-v7.0_LOC_Os01g27880.1                         EAMVPDDGWE EYLNNKWDRN VVVEETAKFS EL-KLQPETE QRPHKVSFFV DKKSAQEVIK SLSGNMEKCG LDVKIIYSGG
O.sativa-v7.0_LOC_Os02g05030.1                         EEMVPDDGWV EYLNNKWDRN IVVEETANVS EL-KLQVESE QRPHKVSFYV DKKSAQEVIK SLSEKLEKRG LDVKIIYSGG
O.sativa-v7.0_LOC_Os05g05270.1                         EEMIHDVGWA EFLSNKWDRD IVVEETAKFP KL-KPQPERS QGPHKVSFFV DKEGAREVMD SLPETLNRRG LDVKIIFSSG
O.thomaeum-v1.0_Oropetium_20150105_15513A              ---------- ---------- ---------- -------ERS QGPHKVSFFV NKQDAQEVID WLPQKLKKRG LDVKIIYSSG
O.thomaeum-v1.0_Oropetium_20150105_22356A              EDMVPDEGWE EYLNNKWDRK IVVEETSTFS EL-KLQPETE QRPHKVSFFV EKKSAQEVIK SLSEKLEKRG VDAKIIYSGG
P.halliiHAL-v2.1_PhHAL.3G110000.1.p_S6PP.1             EEMIRDVGWE EYLDNRWDRN IVVEETAKFS QL-KPQPERN QGPHKVSFFV GKQGAQEVMD SLPQKLEKRG LDVKIVYSSG
P.halliiHAL-v2.1_PhHAL.3G110100.1.p_S6PP.2             ADMVRDVDWE EYLNSNWDWD IVVEEAAKFP QL-KPQWEND QSPHKVSFLV DKQCAQEVMH ALPQKLQSRG IHVKIIFSYG
P.halliiHAL-v2.1_PhHAL.5G367300.1.p                    EAMVPDDGWE EYLNNKWDRN IVVQETASFS EL-KLQPETE QRPHKVSFLV DKKSAQEVIK SVAEKLEKRG LDAKIIYSGG
P.hallii-v3.1_Pahal.3G115900.1.p_S6PP.1                EEMIRDVGWE EYLDNRWDRN IVVEETAKFS QL-KPQPERN QGPHKVSFFV GKQGAQEVMD SLPQKLEKRG LDVKIVYSSG
P.hallii-v3.1_Pahal.3G116000.1.p_S6PP.2                ADMVRDVDWE EYLNSNWDWD IVVEEAAKFP QL-KPQWEND QSPHKVSFLV DKQCAQEVMH ALPQKLQSRG IHVKIIFSYG
P.hallii-v3.1_Pahal.5G373100.1.p                       EAMVPDDGWE EYLNNKWDRN IVVQETASFS EL-KLQPETE QRPHKVSFLV DKKSAQEVIK SVAEKLEKRG LDAKIIYSGG
P.virgatum-v5.1_Pavir.3KG143200.2.p                    EEMIRDVGWE EYLDNKWDRN IVVEETAKFS QL-KPQPERN QGPHKVSFFV GKQDAQEVMD SLPQKLKKRG LDVKIVYSSG
P.virgatum-v5.1_Pavir.3NG188964.1.p_S6PP.1             EEMIRDVGWE EYLDNKWDRN IVVEETAKFS QL-KPQPERN QGPHKVSFFV GKQGAQEVMD SLPQKLEKRG LDVKIVYSSG
P.virgatum-v5.1_Pavir.3NG189313.1.p_S6PP.2             ADMVHDIDWE EYLNSNWDRD IVVEEAAKFP QL-KPQPKND QSPHKVSFLV DKQCAQEVMH ALPQKLQNRG IHVKIIFSYG
P.virgatum-v5.1_Pavir.5KG244800.2.p                    EAMVPDDGWE EYLNNKWDRN IVVQETANFS KL-KLQPETE QRPHKVSFLV DKKSAQEVIK SVAEKLEKRG LDAKIIYSGG
P.virgatum-v5.1_Pavir.5NG369700.2.p                    EAMVPDDGWE EYLNNKWDRN IVVQETASFS EL-KLQPETE QRPHKVSFLV DKKSAQEVIK SLAEKLEKHG LDAKIIYSGG
S.bicolor_RTx430-v2.1_SbiRTX430.04G160000.1.p          EAMVPDDGWE EYLNNKWDRN IVVEETVGFS EL-KLQPETE QRPHKVSFLV DKKSAQEVIK SVAERLEKRG LDAKIIYSGG
S.bicolor_RTx430-v2.1_SbiRTX430.09G042400.1.p_S6PP.1   EEMIRDVGWE EYLNNKWDRT IVVEETARFP QL-KPQPERN QGPHKVSFFV DKQGAREVMD HLPQKLEKRG LDVKIVYSSG
S.bicolor_RTx430-v2.1_SbiRTX430.09G042500.1.p_S6PP.2   TDMVRDFDWE EHLNRNWDRD IVVEEAAKFP QL-KPEPEKD QGPHKVTFLV DKQGAQEVMH ALPHNLKKRG IHVKIIFSYG
S.bicolorRio-v2.1_SbRio.04G161300.1.p                  EAMVPDDGWE EYLNNKWDRN IVVEETVGFS EL-KLQPETE QRPHKVSFLV DKKSAQEVIK SVAGRLEKRG LDAKIIYSGG
S.bicolorRio-v2.1_SbRio.09G043600.1.p_S6PP.1           EEMIRDVGWE EYLNNKWDRA IVVEETARFP QL-KPQPERN QGPHKVSFFV DKQGAQEVMD HLPQKLEKRG LDVKIVYSSG
S.bicolorRio-v2.1_SbRio.09G043700.1.p_S6PP.2           TDMVRDSDWE EHLNRNWDRD IVVEEAAKFP QL-KPEPEKD QGPHKVTFLV DKQGAQEVMH ALPHNLKKRG IHVKIIFSYG
S.bicolor-v3.1.1_Sobic.004G151800.2.p                  EAMVPDDGWE EYLNNKWDRN IVVEETVGFS EL-KLQPETE QRPHKVSFLV DKKSAQEVIK SVAERLEKRG LDAKIIYSGG
S.bicolor-v3.1.1_Sobic.009G040900.2.p_S6PP.1           EEMIRDVGWE EYLNNKWDRA IVVEETARFP QL-KPQPERN QGPHKVSFFV DKQGAQEVMD HLPQKLEKRG LDVKIVYSSG
S.bicolor-v3.1.1_Sobic.009G041000.1.p_S6PP.2           TDMVRDSDWE EHLNRNWDRD IVVEEAAKFP QL-KPEPEKD QGPHKVTFLV DKQGAQEVMH ALPHNLKKRG IHVKIIFSYG
S.italica-v2.2_Seita.2G386000.1.p                      EAMVPDDGWE EYLNNKWDRN IVVQETASFS EL-KLQPETE QRPHKVSFFV DKKSAQEVIK SLAEKLEKRG LDVKIIYSGG
S.italica-v2.2_Seita.3G059500.1.p_S6PP.1               EEMIRDVGWE EYLDNNWDRN IVVEETAKFS QL-KPQPERN QGPHKVSFFV DKQGAQEVID SLPQKLEKRG LDVKIVYSSG
S.italica-v2.2_Seita.3G059600.1.p_S6PP.2               ADMVHDVDWE EYLNSNWDRD IVVE------ ---------- ---------V EEAA------ --------KG KHQTILF---
S.italica-v2.2_Seita.5G174500.1.p                      EAMVPDDGWE EYLNNKWDRN IVVQETASFS EL-KLQPETE QRPHKVSFFV DKKSAQEVIK SVAKKLDKRG LDAKIIYSGG
S.viridis-v2.1_Sevir.2G396500.2.p                      EAMVPDDGWE EYLNNKWDRN IVVQETASFS EL-KLQPETE QRPHKVSFFV DKKSAQEVIK SLAEKLEKRG LDVKIIYSGG
S.viridis-v2.1_Sevir.3G060400.2.p_S6PP.1               EEMIRDVGWE EYLDNNWDRN IVVEETAKFS QL-KPQPERN QGPHKVSFFV DKQGAQEVID SLPQKLEKRG LDVKIVYSSG
S.viridis-v2.1_Sevir.5G174500.1.p                      EAMVPDDGWE EYLNNKWDRN IVVQETASFS EL-KLQPETE QRPHKVSFFV DKKSAQEVIK SVAKKLDKRG LDAKIIYSGG
Saccharum-R570_S6PP.1                                  EEMIRDVGWE EYLNNNWDRT IVVEETAGFP QL-KPQPERN QGPHKVSFFV DKQGAQEVMD YLPQKLEKRG LDVKIVYSNG
Saccharum-R570_S6PP.2                                  ADMVRDADWE EHLSSNWDRD I--------- ------PEKD QGPHKVTFLV DNQGAQEVMH ALPHKLKKRG IHVKIIFSYG
Saccharum-R570_S6PP.2D.2                               ADMVRDADWE EHLSSNWDRD I--------- ------PEKD QGPHKVTFLV DNQGAQEVMH ALPHKLKKRG IHVKIIFSYG
Saccharum-R570_S6PP-2D.1                               EEMIRDVGWE EYLNNNWDRT IVVEETAGFP QL-KPQPERN QGPHKVSFFV DKQGAQEVMD YLPQKLEKRG LDVKIVYSNG
T.aestivum-v2.2_Traes_1BS_998F2E806.2                  QEMTPDVGWE EFLNNKWDRD IVVQETATFP QL-KPQPERN QGPHKVSFSV DRQGAQEVIN SLPQKLEQLG LDVKIIYSSG
T.aestivum-v2.2_Traes_1DS_9AE5A76AC.2                  QEMTPDVGWE EFLNNKWDRD IVVQETAKFP QL-KPQPERN QGPHKVSFFV DKQGAQEVIN YLPQKLEQLG LYVKIIYSSG
T.aestivum-v2.2_Traes_5AS_962BCA20C.1                  EAMVPDHGWE EYLNNKWDRN IVLEETAKFS EL-KLQVETE QRPHKVSFLV DKKNAQGVIK SLSEKFEKRG VDAKIIYSGG
T.aestivum-v2.2_Traes_5BS_F7853DBB6.2                  EAMVPDHGWE EYLNNKWDRN IVLEETAKFS EL-KLQVETE QRPHKVSFLV DKKNAQGVIK SLSETFEKRG VDAKIIYSGG
T.aestivum-v2.2_Traes_5DS_0B17FFD55.1                  EAMVPDHGWE EYLNNKWDRN IVLEETAKFS EL-KLQVESE QRPHKVSFLV DKKNAQGVIK SLSEKFEKRG VDAKIIYSGG
T.intermedium-v2.1_Thint.01G0102700.1.p                QEMTPDVGWE EFLNIKWDRD IVVQETATFP QL-KPQPERN QGPHKVSFFV DKQGAQEVIN SLPQKLEQLG LDVKIIYSSG
T.intermedium-v2.1_Thint.02G0243300.1.p                QEMTPDVGWE EFLNNKWDRD IVVQETATFP QL-KPQPERN QGPHKVSFFV DKQGAQEVIN SLPQKLEQLG LDVKIIYSSG
T.intermedium-v2.1_Thint.03G0206200.1.p                QEMTPDVGWE EFLNNKWDRD IVVQETATFP QL-KPQPERN QGPHKVSFFV DKQGAQEVIN SLPQKLEQLG LDVKIIYSSG
T.intermedium-v2.1_Thint.05G0032000.1.p                EAMIPDDGWE EYLNNKWDRN IVLEETAKFS EL-KLQPETE QRPHKVSFLV DKKSAQEVIK SLSEKFEKRG VDAKIIYSGG
T.intermedium-v2.1_Thint.13G0028000.1.p                EAMVPDDGWE EYLNNKWDKN IVLEETAKFS EL-KLQAETE QRPHKVSFLV DKKNAQGVIK SLSEKFEKRG VDAKIIYSGG
T.intermedium-v2.1_Thint.14G0034900.1.p                EAMVPDHGWE EYLNNKWDRN IVLEETAKFS EL-KLQVETE QRPHKVSFLV DKKNAQGVIK SLSEKFEKRG VDAKIIYSGG
T.intermedium-v2.1_Thint.15G0012900.1.p                EAMVPDHGWE EYLNNKWDRN IVLEETAKFS EL-KLQVETE QRPHKVSFLV DKKNAQGVIK SLSEKFEKRG VDAKIIYSGG
T.intermedium-v2.1_Thint.V1674200.1.p                  EAMIPDDGWE EYLNNKWDRN IVLEETAKFS EL-KLQPETE QRPHKVSFLV DKKSAQEVIK SLSEKFEKRG VDAKIIYSGG
Z.maysPHJ40-v1.1_ZmPHJ40.08G139200.1.p                 EAMVPDDGWE EYLNNKWDRN IVVAETVSFS EL-KLQPETE QRPHKVSFFV DKKNAQEVIK SVAERLDKCG LDAKIIYSGG
Z.maysPHJ40-v1.1_ZmPHJ40.10G092800.1.p                 EEMVRDVGWE ERLDSDWDRA IVVEETARLP QLARMQPERN QGPHKVSFFV DKQGAQEVMD YLPKKLERRG VDVKIVYSSG
```

```
                                                        161
B.distachyon-v3.2_Bradi1g55300.2.p                      QDLDILAQGA GKGQALAYLL KKLGSCGKTP NNTLVCGDSG NDAELFSIPG VHGVMVSNAQ EELLQWRAEN AKDNPKVIHA
B.distachyon-v3.2_Bradi2g36350.6.p                      EALDVLPRGA GKGQALVYLL EKFNSCGKPP NNVLVCGDSG NDADLFSVPS AHGVMVSNAQ EELLQWYEEK GKDNSKMIHA
B.hybridum-v1.1_Brahy.D01G0746500.1.p                   QDLDILAQGA GKGQALAYLL KKLGSCGKTP NNTLVCGDSG NDAELFSIPG VHGVMVSNAQ EELLQWRAEN AKDNPKVIHA
B.hybridum-v1.1_Brahy.D02G0478800.1.p                   EALDVLPRGA GKGQALVYLL EKFNSCGKPP NNVLVCGDSG NDADLFSVPS AHGVMVSNAQ EELLQWYEEK GKDNSKMIHA
B.hybridum-v1.1_Brahy.S06G0207800.1.p                   QDLDILAQGA GKGQALAYLL KKLGSCGKTP SNTLVCGDSG NDAELFSIPG VHGVMVSNAQ EELLQWREEN AKDNPKVIHA
B.hybridum-v1.1_Brahy.S08G0229700.1.p                   EALDILPQGA GKGQALVYLL EKFNSCGKPP NNVLVCGDSG NDADLFSVPS AHGVMVSNAQ EELLQWYEEK GKDNPKIIHA
B.stacei-v1.1_Brast06G189100.1.p                        QDLDILAQGA GKGQALAYLL KKLGSCGKTP SNTLVCGDSG NDAELFSIPG VHGVMVSNAQ EELLQWREEN AKDNPKVIHA
B.stacei-v1.1_Brast08G210900.1.p                        EALDILPQGA GKGQALVYLL EKFNSCGKPP NNVLVCGDSG NDADLFSVPS AHGVMVSNAQ EELLQWYEEK GKDNPKIIHA
B.sylvaticum-v1.1_Brasy6G197100.1.p                     QDLDILAQGA GKGQALAYLL KKLGSCGKTP NNTLVCGDSG NDAELFSIPG VHGVMVSNAQ EELLQWRAEN AKDNPKVIHA
B.sylvaticum-v1.1_Brasy8G226900.1.p                     EALDVLPRGA GKGQALVYLL EKFNSCGKPP NNVLVCGDSG NDADLFSVPS AHGVMVSNAQ EELLQWYEEK GKDNSKMIHA
E.coracana-v1.1_ELECO.r07.1AG0017250.1                  QDLDVLAQGA GKGQALAYLL KKLNSCGKPP NNTLVCGDSG NDAELFSIPG VHGVMVSNAQ EELLQWHADN AKDNPKIIHA
E.coracana-v1.1_ELECO.r07.1BG0066280.1                  QDLDVLAQGA GKGQALAYLL KKLNSCGKPP NNTLVCGDSG NDAELFSIPG VHGVMVSNAQ EELLQWHADN AKDNPKIILA
E.coracana-v1.1_ELECO.r07.5AG0405240.1                  EALDVLPQGA GKGGALSYLL KKFKSQGKPP NNTLVCGDSG NDAELFSVPS AHGVMVSNAQ EELLQWYEEN ARDNDKILHA
E.coracana-v1.1_ELECO.r07.5BG0453340.1                  EALDVLPQGA GKGGALSYLL KKFKSQGKPP NNTLVCGDSG NDAELFSVPS AHGVMVSNAQ EELLQWYEEN ARDNDKILHA
H.vulgare-r1_HORVU1Hr1G019500.3                         EALDVLPQGA GKGQALIYLL EKFNLCGKPP NNVLVCGDSG NDADLFSVPS AYGVMVSNAQ EELLEWYEEN GKDNLKLLYA
H.vulgare-r1_HORVU5Hr1G000580.1                         QDLDILAQGA GKGQALAYLL KKLGSCGKTP NNTLVCGDSG NDAELFSIPG VHGVMVSNAQ EELLQWHAEN AKDNPKIIHA
M.sinensis-v7.1_Misin07G375700.1.p                      QDLDILPQGA GKGQALAYLL KKLSSCGKPP NNTLVCGDSG NDAELFSIPG VHGVMVSNAQ EELLQWYTEN AKDNPKIIHA
M.sinensis-v7.1_Misin16G055500.1.p_S6PP.1               EALDVLPRGA GKGQALMYLL NKLNSYEKPP KNTLVCGDSG NDAELFSVPS VYGVMVCNAQ EELLQWYEEN AKDNPKIIHA
M.sinensis-v7.1_Misin16G055600.1.p_S6PP.2               VLIDVVPQGA GKGQALQYLL NKFNSQRKAP NNILVCGDSG NDAELFSVPS VHGVMVSNAQ EELLQWREEN AMCNPKIIHS
M.sinensis-v7.1_Misin17G051300.1.p_S6PP.2               VLIDVVPQGA GKGQALQYLL NKLNSQRKAP SNILVCGDSG NDAELFSIPS VHGVMVSNAQ EELLQWREEN AMCNPKIIHS
M.sinensis-v7.1_Misin17G051400.1.p_S6PP.1               EAFDVLPRGA GKGQALMYLL NKLNSYEKPP KNTLVCGDSG NDAELFSVPS VYGVMVCNAQ EELLQWYEEN AKDNPKIIHA
O.sativaKitaake-v3.1_OsKitaake01g172900.1.p             QDLDILPQGA GKGQALAYLL KKLSSCGKPP NNTLVCGDSG NDAELFSIPG VHGVMVSNAQ EELLQWYAEN AKGNPKIIHA
O.sativaKitaake-v3.1_OsKitaake02g035100.1.p             QDLDVLPQGA GKGQALAYLL KKLSSCGKPP NNTLACGDSG NDAELFSIPG VHGVMVSNAQ EELLQWYSEN AKDNPKIIHA
O.sativaKitaake-v3.1_OsKitaake05g032900.1.p             EALDVLPQGA GKGQALLYLL KKFNSDGKPP NSTLVCGDSG NDAELFSVPS VHGVMVSNAQ EELLQWYEEN ARGNPMMIHA
O.sativa-v7.0_LOC_Os01g27880.1                          QDLDILPQGA GKGQALAYLL KKLSSCGKPP NNTLVCGDSG NDAELFSIPG VHGVMVSNAQ EELLQWYAEN AKGNPKIIHA
O.sativa-v7.0_LOC_Os02g05030.1                          QDLDVLPQGA GKGQALAYLL KKLSSCGKPP NNTLACGDSG NDAELFSIPG VHGVMVSNAQ EELLQWYSEN AKDNPKIIHA
O.sativa-v7.0_LOC_Os05g05270.1                          EALDVLPQGA GKGQALLYLL KKFNSDGKPP NSTLVCGDSG NDAELFSVPS VHGVMVSNAQ EELLQWYEEN ARGNPMMIHA
O.thomaeum-v1.0_Oropetium_20150105_15513A               EALDVLPQGA GKGQALSYVL KKFNSHGKPP RNTLVCGDSG NDAELFSVPS VHGVMVGNAQ EELLQWYEEN AKDNDKILHA
O.thomaeum-v1.0_Oropetium_20150105_22356A               QDLDVLAEGA GKGQALAYLL KKLNSCGKPP NNTLVCGDSG NDAELFSIPG VHGVMVSNAQ EELLQWYADN AKDNPKIIHA
P.halliiHAL-v2.1_PhHAL.3G110000.1.p_S6PP.1              NALDVLPQGA GKGQALVYLL NTLKSHGKPP NNTLVCGDSG NDAELFSVPS VHGVMVSNAQ EELLQWYEEN AKSNPQIIYA
P.halliiHAL-v2.1_PhHAL.3G110100.1.p_S6PP.2              VLLDVLPQGA GKGQALQYLL NKFSFQGKAP NNTLVCGDSG NDTELFSVPS IHGVMVSNAQ EELLQWYEEN ATYNPKIIHA
P.halliiHAL-v2.1_PhHAL.5G367300.1.p                     QDLDILPQGA GKGQALAYLL KKLSSCGKPP NNTLVCGDSG NDAELFSIPG VHGVMVSNAQ EELLQWYQEN AKDNPKIIHA
P.hallii-v3.1_Pahal.3G115900.1.p_S6PP.1                 NALDVLPQGA GKGQALVYLL NTLKSHGKPP NNTLVCGDSG NDAELFSVPS VHGVMVSNAQ EELLQWYEEN AKSNPQIIYA
P.hallii-v3.1_Pahal.3G116000.1.p_S6PP.2                 VLLDVLPQGA GKGQALQYLL NKFSFQGKAP NNTLVCGDSG NDTELFSVPS IHGVMVSNAQ EELLQWYEEN ATYNPKIIHA
P.hallii-v3.1_Pahal.5G373100.1.p                        QDLDILPQGA GKGQALAYLL KKLSSCGKPP NNTLVCGDSG NDAELFSIPG VHGVMVSNAQ EELLQWYQEN AKDNPKIIHA
P.virgatum-v5.1_Pavir.3KG143200.2.p                     EALDVLPQGA GKGQALVYLL NTLKSHGKAP NNTLVCGDSG NDAELFSVPS VHGVMVSNAQ EELLQWYEEN AKNNPEIIHA
P.virgatum-v5.1_Pavir.3NG188964.1.p_S6PP.1              EALDVLPQGA GKGQALVYLL NTLKSHGKPP NNTLVCGDSG NDAELFSVPS VHGVMVSNAQ EELLQWYEEN AKNNPEIIHA
P.virgatum-v5.1_Pavir.3NG189313.1.p_S6PP.2              VLLDVLPQGA GKGQALQYPL NKFSFQGKAP NNTLVCGDSG NDTELFSVPS IHGVMVSNAQ EELLQWYEEN ATHNPKIIHA
P.virgatum-v5.1_Pavir.5KG244800.2.p                     QDLDILPQGA GKGQALAYLL KKLSSCGKPP NNTLVCGDSG NDAELFSIPG VHGVMVSNAQ EELLQWYQEN AKDNSKIIHA
P.virgatum-v5.1_Pavir.5NG369700.2.p                     QDLDILPQGA GKGQALAYLL KKLSSCGKPP NNTLVCGDSG NDAELFSIPG VHGVMVSNAQ EELLQWYQEN AKDNPKIIHA
S.bicolor_RTx430-v2.1_SbiRTX430.04G160000.1.p           QDLDILPQGA GKGQALAYLL KKLSSCGKPP NNTLVCGDSG NDAELFSIPG VHGVMVSNAQ EELLQWYTEN AKDNPKIIHA
S.bicolor_RTx430-v2.1_SbiRTX430.09G042400.1.p_S6PP.1    EALDVLPQGA GKGQALMYLL NKLNSYEKPP KNTLVCGDSG NDAELFSVPS VHGVMVCNAQ EELLQWYEEN ARDNPKIIHA
S.bicolor_RTx430-v2.1_SbiRTX430.09G042500.1.p_S6PP.2    VLIDVVPQGA GKGQALQYLL NKFTSQRKAP NNILVCGDSG NDAELFSVPS VHGVMVSNAQ EELLQWREEN AMCNPKIIHS
S.bicolorRio-v2.1_SbRio.04G161300.1.p                   QDLDILPQGA GKGQALAYLL KKLSSCGKPP NNTLVCGDSG NDAELFSIPG VHGVMVSNAQ EELLQWYTEN AKDNPKIIHA
S.bicolorRio-v2.1_SbRio.09G043600.1.p_S6PP.1            EALDVLPQGA GKGQALMYLL NKLNSYEKPP KNTLVCGDSG NDAELFSVPS VHGVMVCNAQ EELLQWYEEN ARDNPKIIHA
S.bicolorRio-v2.1_SbRio.09G043700.1.p_S6PP.2            VLIDVVPQGA GKGQALQYLL NKFTLQRKAP NNILVCGDSG NDAELFSVPS VHGVMVSNAQ EELLQWREEN AMCNPKIIHS
S.bicolor-v3.1.1_Sobic.004G151800.2.p                   QDLDILPQGA GKGQALAYLL KKLSSCGKPP NNTLVCGDSG NDAELFSIPG VHGVMVSNAQ EELLQWYTEN AKDNPKIIHA
S.bicolor-v3.1.1_Sobic.009G040900.2.p_S6PP.1            EALDVLPQGA GKGQALMYLL NKLNSYEKPP KNTLVCGDSG NDAELFSVPS VHGVMVCNAQ EELLQWYEEN ARDNPKIIHA
S.bicolor-v3.1.1_Sobic.009G041000.1.p_S6PP.2            VLIDVVPQGA GKGQALQYLL NKFTLQRKAP NNILVCGDSG NDAELFSVPS VHGVMVSNAQ EELLQWREEN AMCNPKIIHS
S.italica-v2.2_Seita.2G386000.1.p                       QDLDILPQGA GKGQALAYLL KKLSSCGKPP NNTLVCGDSG NDAELFSIPD VHGVMVNNAQ EELLQWYTEN AKDNPKIIHA
S.italica-v2.2_Seita.3G059500.1.p_S6PP.1                EALDVLPQGA GKGQALVYLL NTLNSNGKPP NNTLVCGDSG NDAELFSVPS VHGIMVSNAQ EELLQWYEEN AKSNPKIIHA
S.italica-v2.2_Seita.3G059600.1.p_S6PP.2                ---------- ---------- ---------- ----VCGDSG NDTELFSVPS IHGVMVSNAQ EELLQWYEEN ATYNPKIIHS
S.italica-v2.2_Seita.5G174500.1.p                       QDLDILPQGA GKGQALAYLL KKLSSCGKTP NNTLVCGDSG NDAELFSIPD VHGCMVSNAQ EELLQWYQEN AKDNPKIIHA
S.viridis-v2.1_Sevir.2G396500.2.p                       QDLDILPQGA GKGQALAYLL KKLSSCGKPP NNTLVCGDSG NDAELFSIPD VHGVMVNNAQ EELLQWYTEN AKDNPKIIHA
S.viridis-v2.1_Sevir.3G060400.2.p_S6PP.1                EALDVLPQGA GKGQALVYLL NTLNSNGKPP NNTLVCGDSG NDAELFSVPS VHGIMVSNAQ EELLQWYEEN AKSNPKIIHA
S.viridis-v2.1_Sevir.5G174500.1.p                       QDLDILPQGA GKGQALAYLL KKLSSCGKTP NNTLVCGDSG NDAELFSIPD VHGCMVSNAQ EELLQWYQEN AKDNPKIIHA
Saccharum-R570_S6PP.1                                   EALDVLPQGA GKGQALMYLL NKLNSYEKPP KNTLVCGDSG NDAELFSVPS VHGVMVCNAQ EELLQWYEEN ARDNPKIIHA
Saccharum-R570_S6PP.2                                   VLIDVVPQGA GKGQALQYLL NKFTSQGRAP SNILVCGDSG NDAELFSVPS VHGVMVSNAQ EELLQWRKEN AMYNPKIIHS
Saccharum-R570_S6PP.2D.2                                VLIDVVPQGA GKGQALQYLL NKFTSQGRAP SNILVCGDSG NDAELFSVPS VHGVMVSNAQ EELLQWRKEN AMYNPKIIHS
Saccharum-R570_S6PP-2D.1                                EALDVLPQGA GKGQALMYLL NKLNSYEKPP KNTLVCGDSG NDAELFSVPS VHGVMVCNAQ EELLQWYEEN ARDNPKIIHA
T.aestivum-v2.2_Traes_1BS_998F2E806.2                   EALDVLPRGA GKGQALIYLL EKFSSCAKSP NNVLVCGDSG NDADLFSVPS AYGVMVSNAQ EELLQWYEEN GKDKPKLLHA
T.aestivum-v2.2_Traes_1DS_9AE5A76AC.2                   EALDVLPRGA GKGQALIYLL EKFSSSGKPP SNVLVCGDSG NDADLFSVPS AYGVMVSNAQ EELLQWYEEN GKDNLKLLHA
T.aestivum-v2.2_Traes_5AS_962BCA20C.1                   QDLDILAQGA GKGQALAYLL KKLGSCGKTP NNTLACGDSG NDAELFSIPG VHGVMVSNAQ EELLQWHAEN AKDNPKIIHA
T.aestivum-v2.2_Traes_5BS_F7853DBB6.2                   QDLDILAQGA GKGQALAYLL KKLGSCGKTP NNTLACGDSG NDAELFSIPG VHGVMVSNAQ EELLQWHAEN AKDNPKIIHA
T.aestivum-v2.2_Traes_5DS_0B17FFD55.1                   QDLDILAQGA GKGQALAYLL KKLGSCGKTP NNTLACGDSG NDAELFSIPG VHGVMVSNAQ EELLQWHAEN AKDNPKIIHA
T.intermedium-v2.1_Thint.01G0102700.1.p                 EALDVLPQGA GKGQALIYLL EKFSSCGKPP NNVLVCGDSG NDADLFSVPS AYGVMVSNAQ EELLQWYEES GKGNLKLLYA
T.intermedium-v2.1_Thint.02G0243300.1.p                 EALDVLPRGA GKGQALVYLL EKFSSCGKPP NNVLVCGDSG NDADLFSVPS AYGVMVSNAQ EELLQWYEEN GKDNLKLLHA
T.intermedium-v2.1_Thint.03G0206200.1.p                 EALDVLPRGA GKGQALIYLL EKFSSCGKPP SNVLVCGDSG NDADLFSVPS AYGVMVSNAQ EELLQWYEEN GKDNLKLLYA
T.intermedium-v2.1_Thint.05G0032000.1.p                 QDLDILAQGA GKGQALAYLL KKLSLCGKTP NNTLVCGDSG NDAELFSIPG VHGVMVSNAQ EELLQWHAEN AKDNPKVIHA
T.intermedium-v2.1_Thint.13G0028000.1.p                 QDLDILAQGA GKGQALAYLL KKLGSCGKTP NNTLACGDSG NDAELFSIPG VHGVMVSNAQ EELLQWHAEN AKDNPKIIHA
T.intermedium-v2.1_Thint.14G0034900.1.p                 QDLDILAQGA GKGQALAYLL KKLGSCGKTP NNTLACGDSG NDAELFSIPG VHGVMVSNAQ EELLQWHAEN AKDNPKIIHA
T.intermedium-v2.1_Thint.15G0012900.1.p                 QDLDILAQGA GKGQALAYLL KKLGSCGKTP TNTLACGDSG NDAELFSIPG VHGVMVSNAQ EELLQWHAEN AKDNPKIIHA
T.intermedium-v2.1_Thint.V1674200.1.p                   QDLDILAQGA GKGQALAYLL KKLSLCGKTP NNTLVCGDSG NDAELFSIPG VHGVMVSNAQ EELLQWHAEN AKDNPKVIHA
Z.maysPHJ40-v1.1_ZmPHJ40.08G139200.1.p                  QDLDILPQGA GKGQALAYLL EKLSSCGKPP NNTLVCGDSG NDAELFSIPG VHGVMVSNAQ EELLQWYTEN AKDNPKIIHS
Z.maysPHJ40-v1.1_ZmPHJ40.10G092800.1.p                  NALDVLPRGA GKGQALVYLL KKLDSNGKPP KNTLVCGDSG NDAELFGVPS VHGVMVCNAQ EELVQWYQEN ARDNPRIIQA
```

```
                                                      241
B.distachyon-v3.2_Bradi1g55300.2.p                     TERCAAGIIQ AIGHFKL
B.distachyon-v3.2_Bradi2g36350.6.p                     TERCASGIIQ AIGHFKL
B.hybridum-v1.1_Brahy.D01G0746500.1.p                  TERCAAGIIQ AIGHFKL
B.hybridum-v1.1_Brahy.D02G0478800.1.p                  TERCASGIIQ AIGHFKL
B.hybridum-v1.1_Brahy.S06G0207800.1.p                  TERCAAGIIQ AIGHFKL
B.hybridum-v1.1_Brahy.S08G0229700.1.p                  TERCASGIMQ AIGHFKL
B.stacei-v1.1_Brast06G189100.1.p                       TERCAAGIIQ AIGHFKL
B.stacei-v1.1_Brast08G210900.1.p                       TERCASGIMQ AIGHFKL
B.sylvaticum-v1.1_Brasy6G197100.1.p                    TERCAAGIIQ AIGHFKL
B.sylvaticum-v1.1_Brasy8G226900.1.p                    TERCASGIIQ AIGHFKL
E.coracana-v1.1_ELECO.r07.1AG0017250.1                 TERCAAGIIQ AIGHFKL
E.coracana-v1.1_ELECO.r07.1BG0066280.1                 TERCAAGIIQ AIGHFKL
E.coracana-v1.1_ELECO.r07.5AG0405240.1                 TERCAAGIMQ AIGHFNL
E.coracana-v1.1_ELECO.r07.5BG0453340.1                 TERCAAGIMQ AIGHFNL
H.vulgare-r1_HORVU1Hr1G019500.3                        TERCASGIMQ AIGHFKL
H.vulgare-r1_HORVU5Hr1G000580.1                        TERCAAGIIQ AIGHFKL
M.sinensis-v7.1_Misin07G375700.1.p                     NDRCAAGIIQ AIGHFKL
M.sinensis-v7.1_Misin16G055500.1.p_S6PP.1              TERCAAGIMQ AIGHFKL
M.sinensis-v7.1_Misin16G055600.1.p_S6PP.2              TKRCAAGIMQ AIGHFKL
M.sinensis-v7.1_Misin17G051300.1.p_S6PP.2              TKRCAAGIMQ AIAHFKL
M.sinensis-v7.1_Misin17G051400.1.p_S6PP.1              TERCAAGIMQ AIGHFKL
O.sativaKitaake-v3.1_OsKitaake01g172900.1.p            TERCAAGIIE AIGHFKL
O.sativaKitaake-v3.1_OsKitaake02g035100.1.p            TERCAAGIIQ AIGHFKL
O.sativaKitaake-v3.1_OsKitaake05g032900.1.p            TERCAAGIMQ AIGHFNL
O.sativa-v7.0_LOC_Os01g27880.1                         TERCAAGIIE AIGHFKL
O.sativa-v7.0_LOC_Os02g05030.1                         TERCAAGIIQ AIGHFKL
O.sativa-v7.0_LOC_Os05g05270.1                         TERCAAGIMQ AIGHFNL
O.thomaeum-v1.0_Oropetium_20150105_15513A              TDRCAAGIMQ AIGHFNL
O.thomaeum-v1.0_Oropetium_20150105_22356A              TERCAAGIIQ AIGHFKL
P.halliiHAL-v2.1_PhHAL.3G110000.1.p_S6PP.1             TERCAAGILQ AIGHFKL
P.halliiHAL-v2.1_PhHAL.3G110100.1.p_S6PP.2             TKRCAAGIME AIGHFKL
P.halliiHAL-v2.1_PhHAL.5G367300.1.p                    SERCAAGIIQ AIGHFKL
P.hallii-v3.1_Pahal.3G115900.1.p_S6PP.1                TERCAAGIMQ AIGHFKL
P.hallii-v3.1_Pahal.3G116000.1.p_S6PP.2                TKRCAAGIME AIGHFKL
P.hallii-v3.1_Pahal.5G373100.1.p                       SERCAAGIIQ AIGHFKL
P.virgatum-v5.1_Pavir.3KG143200.2.p                    TERCAAGIMQ AIGHFKL
P.virgatum-v5.1_Pavir.3NG188964.1.p_S6PP.1             TERCAAGIMQ AIGHFKL
P.virgatum-v5.1_Pavir.3NG189313.1.p_S6PP.2             TKRCAAGIME AIGHFKL
P.virgatum-v5.1_Pavir.5KG244800.2.p                    SERCAAGIIQ AIGHFKL
P.virgatum-v5.1_Pavir.5NG369700.2.p                    SERCAAGIIQ AIGHFKL
S.bicolor_RTx430-v2.1_SbiRTX430.04G160000.1.p          NERCAAGIIQ AIGHFKL
S.bicolor_RTx430-v2.1_SbiRTX430.09G042400.1.p_S6PP.1   TERCATGIMQ AIGHFKL
S.bicolor_RTx430-v2.1_SbiRTX430.09G042500.1.p_S6PP.2   TKRCAAGVMQ AIGHFKL
S.bicolorRio-v2.1_SbRio.04G161300.1.p                  NERCAAGIIQ AIGHFKL
S.bicolorRio-v2.1_SbRio.09G043600.1.p_S6PP.1           TERCAAGIMQ AIGHFKL
S.bicolorRio-v2.1_SbRio.09G043700.1.p_S6PP.2           TKRCAAGVMQ AIGHFKL
S.bicolor-v3.1.1_Sobic.004G151800.2.p                  NERCAAGIIQ AIGHFKL
S.bicolor-v3.1.1_Sobic.009G040900.2.p_S6PP.1           TERCAAGIMQ AIGHFKL
S.bicolor-v3.1.1_Sobic.009G041000.1.p_S6PP.2           TKRCAAGVMQ AIGHFKL
S.italica-v2.2_Seita.2G386000.1.p                      SERCAAGIIQ AIGHFKL
S.italica-v2.2_Seita.3G059500.1.p_S6PP.1               TERCAAGIMQ AIGHFKL
S.italica-v2.2_Seita.3G059600.1.p_S6PP.2               MKRCAAGIME AIEHFKL
S.italica-v2.2_Seita.5G174500.1.p                      SERCAAGIIQ AIGHFKL
S.viridis-v2.1_Sevir.2G396500.2.p                      SERCAAGIIQ AIGHFKL
S.viridis-v2.1_Sevir.3G060400.2.p_S6PP.1               TERCAAGIMQ AIGHFKL
S.viridis-v2.1_Sevir.5G174500.1.p                      SERCAAGIIQ AIGHFKL
Saccharum-R570_S6PP.1                                  TERCAAGIMQ AIGHFKL
Saccharum-R570_S6PP.2                                  TKRCAAGIMQ AIGNFKL
Saccharum-R570_S6PP.2D.2                               TKRCAAGIMQ AIGNFKL
Saccharum-R570_S6PP-2D.1                               TERCAAGIMQ AIGHFKL
T.aestivum-v2.2_Traes_1BS_998F2E806.2                  TERCASGIMQ TIGHFKL
T.aestivum-v2.2_Traes_1DS_9AE5A76AC.2                  TERCASGIMQ AIGHFKL
T.aestivum-v2.2_Traes_5AS_962BCA20C.1                  TERCAAGIIQ AIGHFKL
T.aestivum-v2.2_Traes_5BS_F7853DBB6.2                  TERCAAGIIQ AIGHFKL
T.aestivum-v2.2_Traes_5DS_0B17FFD55.1                  TERCAAGIIQ AIGHFKL
T.intermedium-v2.1_Thint.01G0102700.1.p                TERCASGIMQ AIGHFKL
T.intermedium-v2.1_Thint.02G0243300.1.p                TERCASGIMQ AIGHFKL
T.intermedium-v2.1_Thint.03G0206200.1.p                TERCASGIMQ AIGHFKL
T.intermedium-v2.1_Thint.05G0032000.1.p                TERCAAGIIQ AIGHFKL
T.intermedium-v2.1_Thint.13G0028000.1.p                TERCAAGIIQ AIGHFKL
T.intermedium-v2.1_Thint.14G0034900.1.p                TERCAAGIIQ AIGHFKL
T.intermedium-v2.1_Thint.15G0012900.1.p                TERCAAGIIQ AIGHFKL
T.intermedium-v2.1_Thint.V1674200.1.p                  TERCAAGIIQ AIGHFKL
Z.maysPHJ40-v1.1_ZmPHJ40.08G139200.1.p                 NERCAAGIIQ AIGHFKL
Z.maysPHJ40-v1.1_ZmPHJ40.10G092800.1.p                 TERCAAGIMQ AIGHLGL
```
