# Supplementary material for: Sucrose-phosphate phosphatase from sugarcane reveals an ancestral tandem duplication
Source: BMC Plant Biol. 2021 Jan 7;21:23. doi: 10.1186/s12870-020-02795-5 (PMC7792115; doi:10.1186/s12870-020-02795-5)
Supplement: Supplementary file 5 — Additional file 5: Supplmentary Figure 5. S6PP dNdS from sugarcane and close relatives. [file 12870_2020_2795_MOESM5_ESM.pdf]

	dN/dS	Synonymous substitution rate	Non-synonymous substitution rate
S6PP.1 x S6PP.2	0.2450	d_S = 0.5421	*dN = 0.1328*
S6PP.1 x S6PP2D	0.2450	d_S = 0.5421	d_N = 0.1328
S6PP.1 x S6PP.2_Misin17G051300.1	0.2507	d_S = 0.5520	d_N = 0.1384
S6PP.1 x S6PP.1_Misin17G051400.	0.1612	d_S = 0.0887	d_N = 0.0143
S6PP.1 x Misin07G375700.1_ZmSPP1	0.1294	d_S = 1.1537	d_N = 0.1492
S6PP.2 x S6PP2D	0.0010	d_S = 0.0000	d_N = 0.0000
S6PP.2 x S6PP.2_Misin17G051300.1	0.1973	d_S = 0.1369	d_N = 0.0270
S6PP.2 x S6PP.1_Misin17G051400.	0.2442	d_S = 0.5728	d_N = 0.1399
S6PP.2 x Misin07G375700.1_ZmSPP1	0.1587	d_S = 1.2224	d_N = 0.1940
S6PP_2D (1st domain) x S6PP.1	0.0010	d_S = 0.0000	d_N = 0.0000
S6PP_2D (1st domain) x S6PP2D	0.2450	d_S = 0.5421	d_N = 0.1328
S6PP.1_Misin17G051400.1 x S6PP.1_Misin16G055600.1	0.2577	d_S = 0.5579	d_N = 0.1438
S6PP.1_Misin17G051400.1 x S6PP.2_Misin17G051300.1	0.2441	d_S = 0.5848	d_N = 0.1427
S6PP.1_Misin17G051400.1 x Misin07G375700.1_ZmSPP1	0.1347	d_S = 1.0969	d_N = 0.1477
ZmSPP108G139200.1 x Misin07G375700.1_ZmSPP1	0.2964	d_S = 0.1489	d_N = 0.0441
ZmSPP108G139200.1 x ZmSPP210G092800.1	0.0808	d_S = 2.9292	d_N = 0.2368

PAL2NAL calculations performed with nucleotide and corresponding amino acids from sucrose-6F-phosphatase phosphohydrolase domain presented in figure 4
